# Cross-cohort generalization limitations in gut microbiome-based Alzheimer’s disease classifiers: evidence from four supervised cohorts with batch-correction analysis

**DOI:** 10.3389/fnagi.2026.1918251

**Published:** 2026-10-22

**Authors:** Arhan Chakravarthy

**Affiliations:** Independent Researcher, Cupertino, CA, United States

**Keywords:** 16S rRNA sequencing, Alzheimer’s disease, batch correction, cross-cohort generalization, gut microbiome, machine learning, PERMANOVA, SHAP

## Abstract

Gut microbiome dysbiosis has been proposed as an Alzheimer’s disease (AD) biomarker, but the transportability of microbiome classifiers across independent cohorts remains uncertain. We reprocessed five public 16S rRNA cohorts (509 samples, China, Kazakhstan, South Korea) with a common DADA2/genus-level pipeline and evaluated logistic regression and LightGBM classifiers using nested within-cohort cross-validation, leave-one-cohort-out (LOCO) transfer, pairwise transfer, permutational multivariate analysis of variance (PERMANOVA), batch adjustment, and out-of-fold SHapley Additive exPlanations (SHAP) analyses. Within four labeled cohorts, using strict training-only genus selection as the primary classifier-performance pipeline (Section 2.5.1), within-cohort area under the receiver operating characteristic curve (AUC-ROC) ranged from 0.611 to 0.996. The near-perfect Li 2019 result was stable across 50 repeated nested-cross-validation (CV) partitions and exceeded a 1,000-permutation label null (*p* = 0.000999), though a technical metadata confound cannot be excluded (Section 4.3). In LOCO evaluation, AUC ranged from 0.506 to 0.812 (mean decreases of 0.170 for logistic regression and 0.133 for LightGBM); paired bootstrap intervals excluded zero for four of eight cohort–model differences. LOCO estimates agreed closely with a fixed common-universe reference retained for secondary analyses (max |Δ| = 0.066), though one pairwise-transfer cell changed materially (Kazakhstan→Ling 2020, LightGBM: 0.58 → 0.44), reported without smoothing. In the four-cohort marginal PERMANOVA model, cohort-associated compositional variation exceeded diagnosis-associated variation (R^2^ = 0.172 vs. 0.014), decreasing to 0.059 vs. 0.023 when restricted to three more technically comparable Chinese paired-end MiSeq cohorts; dispersion differed significantly among cohorts. Label-blind transductive ComBat-seq and MMUPHin adjustment changed mean LOCO AUC by at most 0.007 and did not recover transferability. Cross-cohort overlap of top predictive taxa was low: logistic regression overlap remained above a prevalence-restricted null, while LightGBM did not. A descriptive coefficient-stability screen identified eight logistic-regression genera with opposite, stable fitted-coefficient directions across cohorts. These findings demonstrate substantial, heterogeneous cross-cohort generalization limitations among the evaluated public 16S datasets and show that, under the tested label-blind transductive design, the two batch-adjustment methods did not restore transferability. Prospective validation in larger, geographically and technically diverse cohorts is needed before microbiome-based AD classifiers can be considered clinically actionable.

## 1 Introduction

Alzheimer’s disease (AD) is the most common cause of dementia, accounting for an estimated 60–70% of cases; approximately 57 million people worldwide were living with dementia in 2021 (World Health Organization, 2026). The gut–brain axis has emerged as a mechanistic pathway linking intestinal microbial communities to neurodegeneration: altered gut microbiome composition has been associated with AD pathology through neuroinflammatory, metabolic, and neuroendocrine routes (Cryan et al., 2019; Kowalski and Mulak, 2019). Since the landmark observation that germ-free APPPS1 transgenic mice showed reduced amyloid-β pathology in the absence of gut microbiota compared with conventionally colonized controls (Harach et al., 2017), dozens of 16S rRNA and shotgun metagenomic studies have reported significant differences in gut microbial composition between AD or mild cognitive impairment (MCI) patients and cognitively normal (CN) controls. MCI is included as a diagnostic category because it can represent a preclinical or prodromal stage of AD, though not all patients with MCI progress to AD, and the primary supervised analyses here focus on binary AD versus CN comparisons (Section 2.4).

The clinical appeal of a gut microbiome-based diagnostic signature is considerable: stool collection is non-invasive and inexpensive, and microbiome profiling is increasingly accessible. Multiple independent groups have trained machine learning classifiers on gut microbiome abundance profiles and reported promising within-cohort discrimination, with area under the receiver operating characteristic curve (AUC-ROC) values of 0.70–0.90 in several published studies (Zhuang et al., 2018; Ling et al., 2021; Zhu et al., 2022). A critical property of any diagnostic biomarker—transferability across independent populations—has rarely been tested in this literature.

The limited cross-cohort comparisons that do exist reveal a striking inconsistency in the direction of reported associations. A systematic review and meta-analysis synthesizing 17 published studies (679 AD/MCI patients, 632 controls) documented that *Bacteroides* abundance was significantly elevated in North American cohorts yet significantly reduced in Chinese cohorts—a directional reversal across geographically distinct populations (Jemimah et al., 2023). Similar contradictions have been observed for *Akkermansia*, *Faecalibacterium*, and *Lactobacillus* (Vogt et al., 2017; Liu et al., 2019; Zhuang et al., 2018), suggesting that published within-cohort classifiers may detect cohort-specific compositional signatures—driven by diet, geography, host genetics, sequencing protocols, or a combination—rather than a universal microbial correlate of AD pathology.

A recent multi-cohort study integrating 15 public AD microbiome projects identified candidate gut microbiota indicators across multiple geographic populations (Wang et al., 2026), confirming that multi-population integration remains a methodological frontier. The present study is complementary, systematically quantifying cross-cohort generalization failure and empirically evaluating whether two commonly used batch-correction approaches recover cross-cohort transferability—questions that remain open even where multi-cohort classifiers have been attempted.

Despite this recognized heterogeneity, few studies have jointly quantified: (1) the magnitude of cross-cohort AUC collapse when trained classifiers are applied to independent cohorts; (2) the relative magnitude of cohort- versus diagnosis-associated compositional variance, as estimated by variance partitioning—noting that “cohort” is itself a composite, study-of-origin variable (bundling sequencing platform, library preparation, recruitment site, diagnostic ascertainment, geography, and diet, among other differences) that a method such as permutational multivariate analysis of variance (PERMANOVA) cannot decompose into technical and biological components; and (3) whether the two evaluated batch-adjustment methods can recover cross-cohort generalization. Answering these three questions jointly in one rigorous analysis is the primary contribution of this study.

We assembled five publicly available 16S rRNA gene sequencing cohorts spanning three countries (China, Kazakhstan, South Korea): four with per-sample diagnosis labels supporting supervised classification, and a fifth (Kim/KBASE 2022) contributing only to unsupervised compositional analyses (Section 2.1). We conducted a comprehensive cross-cohort generalization evaluation across the four labeled cohorts. We report that classifiers showed substantial and variable degradation when transferred to independent cohorts; cohort identity explains substantially more compositional variance than disease status, though the size of this disparity depends materially on which cohorts are compared; under label-blind transductive adjustment, ComBat-seq and MMUPHin left mean cross-cohort AUC essentially unchanged and did not recover transferability; and top predictive taxa showed low absolute overlap across cohorts, with a subset showing opposite, stable fitted-coefficient directions. Together, these findings constitute a systematic quantification of cross-cohort generalization limitations among the evaluated public gut microbiome AD classifier datasets, with direct implications for the field’s trajectory toward clinical translation.

## 2 Materials and methods

### 2.1 Cohort identification and data acquisition

We systematically searched National Center for Biotechnology Information Sequence Read Archive (NCBI SRA) and European Nucleotide Archive (ENA) for publicly available 16S rRNA amplicon-sequencing studies of gut microbiota in AD, MCI, or cognitively normal populations deposited before January 2025 (search terms and full inclusion/exclusion record: Supplementary Note S1). Inclusion required per-sample diagnosis labels, raw or quality-filtered reads in a public archive, and ≥40 participants per cohort; shotgun metagenomic studies were excluded to avoid mixing amplicon and whole-genome platforms, and cohorts were excluded for Institutional Review Board (IRB)-restricted labels, unreleased data, or a non-fecal tissue compartment.

Four cohorts met all criteria for supervised analysis. A fifth, Kim/KBASE 2022 (Jung et al., 2022; PRJEB50447; 78 participants, 18 preclinical AD by amyloid-PET, 60 CN), lacked publicly available per-sample diagnosis labels and was retained only for PERMANOVA (Phase 4) and excluded from all supervised classifier training and evaluation. The ALBION 2025 Greek cohort (PRJNA1297934) was identified but excluded because data had not yet been publicly released at the time of analysis.

The four cohorts with per-sample diagnosis labels used in supervised analyses were:

*Zhuang 2018 (China; PRJNA554111)*: 86 participants (43 AD, 43 CN) from the Third Military Medical University, Chongqing. Paired-end MiSeq 2 × 300 bp, V3–V4 region (Zhuang et al., 2018).
*Ling 2020 (China; PRJNA633959)*: 171 participants (100 AD, 71 CN). Paired-end MiSeq 2 × 300 bp, V3–V4 region (Ling et al., 2021).
*Li 2019 (China; PRJNA489760)*: The SRA deposit contains 180 samples representing 90 participants (30 AD, 30 MCI, 30 CN), each contributing two samples: a fecal (F_) sample and a blood microbiome (B_) sample. Only the 90 fecal samples were used in all analyses; blood samples were excluded as a distinct biological compartment that would confound within-cohort classifiers. Binary analyses used 30 AD and 30 CN fecal samples (*n* = 60). Paired-end MiSeq 2 × 300 bp, V3 -V4 (Li et al., 2019). Diagnostic ascertainment differed across cohorts (Zhuang 2018/Ling 2020/Li 2019: clinical AD diagnosis; Kazakhstan: NINCDS-ADRDA criteria; Kim/KBASE 2022: amyloid-PET positivity); Section 4.6 discusses the implications of this heterogeneity.
*Kazakhstan 2022 (PRJNA811324)*: 84 participants (41 AD, 43 CN). Single-end NovaSeq sequencing, V3–V4 region (Kaiyrlykyzy et al., 2022)—the only single-end dataset in the study (Section 2.2, Limitations).

All raw FASTQ data were downloaded from NCBI SRA using fasterq-dump (SRA Toolkit v3.0.10). Cohort-level sequencing statistics are reported in Supplementary Table S2.

### 2.2 DADA2 amplicon sequence variant processing

Raw paired-end reads (four cohorts) and single-end reads (Kazakhstan) were processed independently per cohort using DADA2 v1.28 in R v4.3 (Callahan et al., 2016) to avoid introducing cross-cohort data leakage during error model estimation.

For paired-end cohorts, reads were trimmed to truncation lengths selected per cohort based on per-base quality score profiles (generally 240 bp forward / 200 bp reverse for V3–V4 MiSeq 2 × 300 data), filtered to remove reads with >2 expected errors, were subjected to chimera removal using the consensus method, and merged. For Kazakhstan (single-end), the single-end DADA2 workflow was applied without merging; the truncation length was adjusted to 250 bp after inspection of quality profiles (Supplementary Note S2). The mixed single-end/paired-end design is an additional technical source of between-cohort heterogeneity, acknowledged as a study limitation.

Taxonomic assignment used the SILVA reference database v138.1. amplicon sequence variants (ASV) were collapsed to the genus level by summing raw counts per sample. Two genus-selection schemes were used in this study, and every downstream result states which one it uses (Section 2.5.1 gives the full rationale). The primary scheme, used for every headline classifier-performance estimate (within-cohort, LOCO, pairwise transfer; Tables 1, 2; Figures 1, 2), re-derives the ≥20%-prevalence genus set independently within each train/test split, from training-side samples only, so no held-out cohort ever contributes prevalence information to its own evaluation. A fixed common-universe scheme— ≥ 20% prevalence (non-zero count in ≥20% of samples) in at least one of the five cohorts, computed once globally and pooling all five cohorts’ raw prevalence, producing a fixed 396-genus feature set—is retained only for the secondary descriptive/harmonization analyses that require one common feature space across cohorts simultaneously (batch correction, SHAP, PERMANOVA; Section 2.5.1). Genera absent in a given sample received a count of zero under either scheme.

**Table 1:** Within-cohort nested cross-validation performance under strict training-only genus selection (10-fold outer, 5-fold inner CV; primary analysis, Section 2.5.1).

Cohort	Model	*N*	AUC-ROC	95% CI	Sensitivity	Specificity	N genera (range)	Originally reported AUC
Zhuang 2018	LogReg	86	0.665	[0.55–0.78]	0.767	0.581	129–132	~0.73 (Zhuang et al., 2018)
Zhuang 2018	LGBM	86	0.673	[0.56–0.78]	0.721	0.628	129–132	—
Ling 2020	LogReg	171	0.890	[0.84–0.93]	0.750	0.944	125–129	~0.87 (Ling et al., 2021)
Ling 2020	LGBM	171	0.842	[0.78–0.90]	0.720	0.930	125–129	—
Li 2019	LogReg	60	0.996	[0.98–1.00]	0.933	1.000	112–119	—
Li 2019	LGBM	60	0.970	[0.93–1.00]	0.933	0.933	112–119	—
Kaiyrlykyzy 2022	LogReg	84	0.737	[0.62–0.84]	0.732	0.744	342–361	~0.78 (Kaiyrlykyzy et al., 2022)
Kaiyrlykyzy 2022	LGBM	84	0.611	[0.49–0.73]	0.415	0.814	342–361	—

*N* = 60 for Li 2019 binary analysis (30 AD + 30 CN fecal samples; MCI and blood samples were excluded). Genus selection was re-derived independently within every one of the 10 outer folds, using only that fold’s training-side samples (Section 2.5.1); “N Genera (Range)” gives the min–max retained-genus count across those 10 folds, versus 396 under the fixed common-universe schema (Supplementary Table S4). Originally reported AUC values are approximate, derived from the primary publications using the best-performing classifier reported; direct numerical comparison is limited by differences in cohort subset, model type, and validation design. All 95% CIs in this table use the diagnosis-stratified, participant-level paired bootstrap of Section 2.5.2 (10,000 replicates), applied to these training-only out-of-fold predictions. The corresponding common-universe (fixed 396-genus) reference values are given in Supplementary Table S4.

**Table 2:** LOCO cross-cohort AUC-ROC versus within-cohort nested CV baseline, both under strict training-only genus selection (primary analysis, Section 2.5.1).

Test Cohort	Model	Within-Cohort AUC	LOCO AUC	LOCO 95% CI	AUC Drop
Zhuang 2018	LogReg	0.665	0.506	[0.38–0.63]	0.159
Zhuang 2018	LGBM	0.673	0.596	[0.47–0.72]	0.077
Ling 2020	LogReg	0.890	0.679	[0.60–0.76]	0.210
Ling 2020	LGBM	0.842	0.653	[0.57–0.73]	0.188
Li 2019	LogReg	0.996	0.812	[0.69–0.93]	0.183
Li 2019	LGBM	0.970	0.788	[0.66–0.89]	0.182
Kazakhstan	LogReg	0.737	0.609	[0.49–0.73]	0.128
Kazakhstan	LGBM	0.611	0.525	[0.40–0.65]	0.086
Mean	LogReg	0.822	0.652	—	0.170
Mean	LGBM	0.774	0.641	—	0.133

Paired-bootstrap significance for the AUC drop (Section 2.5.2; Supplementary Table S10) indicates that 4 of 8 cohort–model pairs have a 95% CI excluding zero (Ling 2020 and Li 2019, both models). The common-universe reference analysis results in five of eight, differing only in Kazakhstan LogReg: its delta excludes zero under the common-universe schema [0.201, 95% CI (0.04, 0.36)] but not under training-only selection [0.128, 95% CI (−0.03, 0.28)]; full per-pair numbers can be found in Supplementary Table S10.

**Figure 1:**
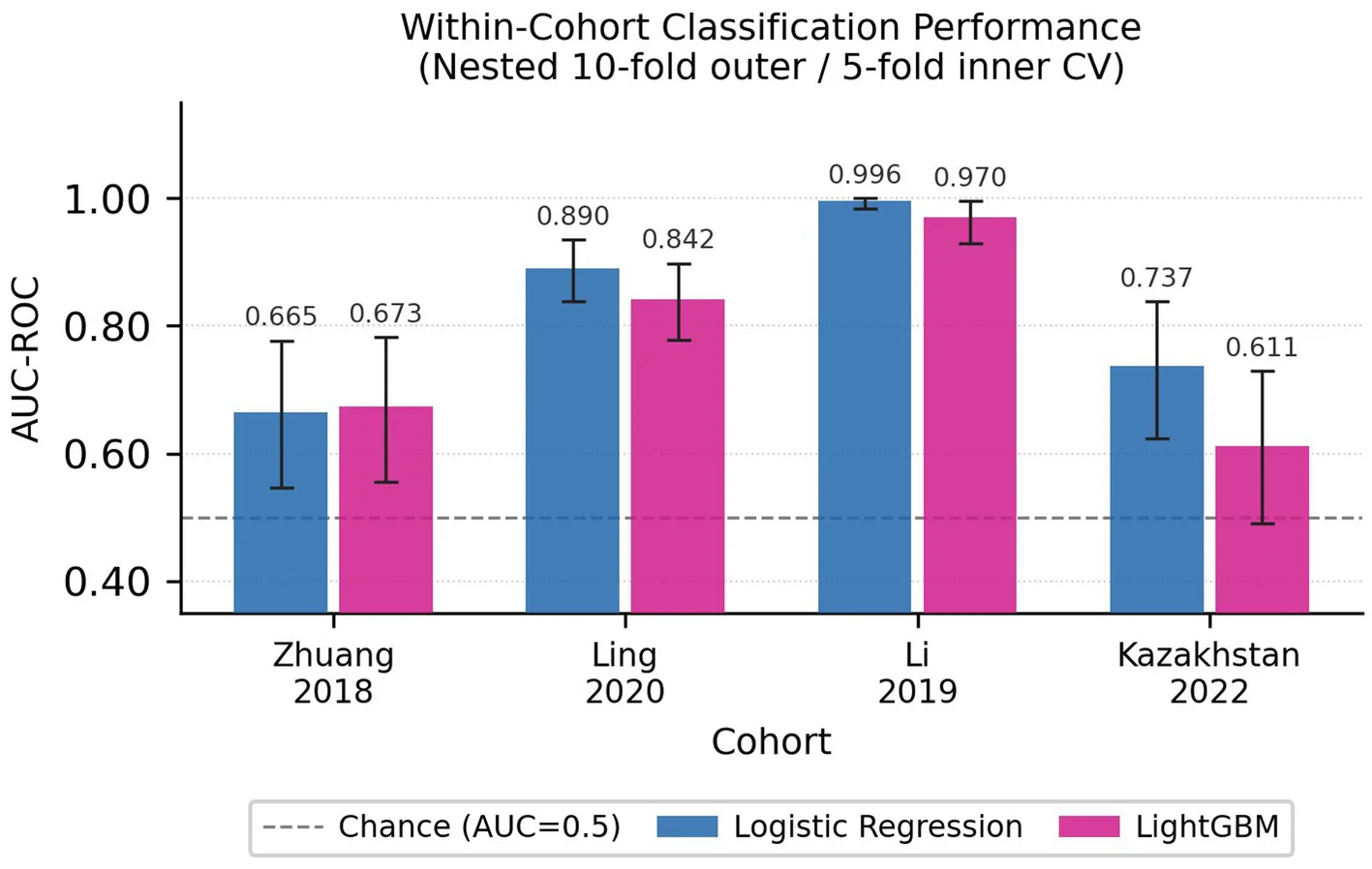
Within-cohort classification performance under nested cross-validation (10-fold outer/5-fold inner CV), using strict training-only genus selection as the primary classifier-performance pipeline (Section 2.5.1). Bars show out-of-fold AUC-ROC; error bars represent 95% CIs from the diagnosis-stratified, participant-level bootstrap (10,000 replicates; Section 2.5.2), matching Table 1. Dashed line = chance (AUC = 0.50). LogReg = L2-regularized logistic regression; LGBM = LightGBM. Matched values under the fixed common-universe (396-genus) schema are provided in Supplementary Table S4.

**Figure 2:**
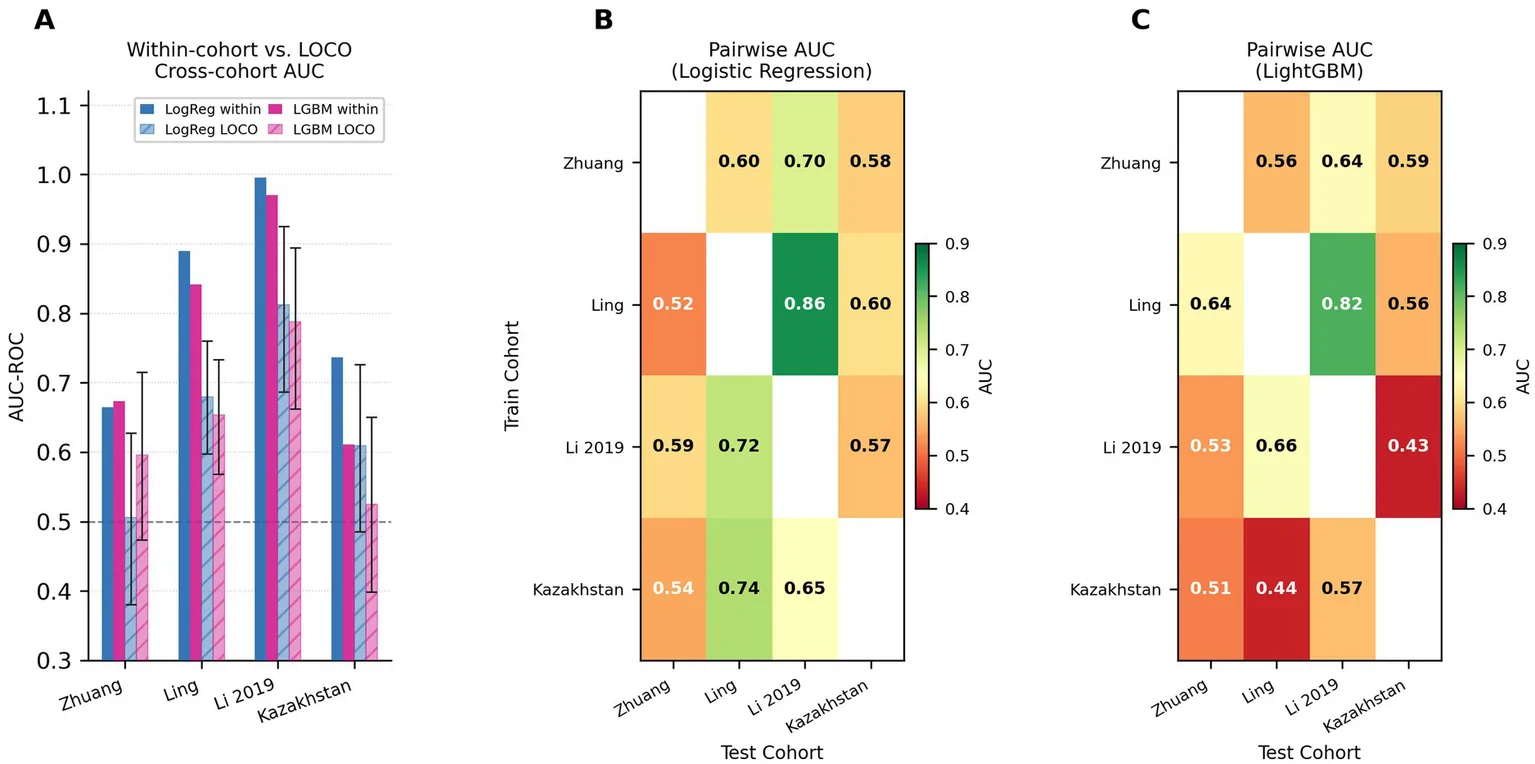
Cross-cohort generalization limitations, using strict training-only genus selection as the primary classifier-performance pipeline (Section 2.5.1). **(A)** Within-cohort (solid bars) versus LOCO cross-cohort (hatched bars) AUC-ROC for logistic regression (blue) and LightGBM (pink). Error bars on the LOCO bars represent the 95% CI from the diagnosis-stratified, participant-level bootstrap (10,000 replicates; Section 2.5.2), matching Table 2; within-cohort bars are shown without error bars in this panel. **(B,C)** Pairwise single-cohort-train / single-cohort-test AUC heatmaps for logistic regression and LightGBM, respectively. Diagonal (within-cohort) values are omitted; color scale: red = lower AUC, green = higher AUC. Matched values under the fixed common-universe (396-genus) schema are provided in Supplementary Table S1; one cell (Kazakhstan→Ling 2020, LightGBM) differs materially between the two pipelines (0.58 vs. 0.44) and is discussed in Section 3.3.

#### 2.2.1 Leakage considerations

DADA2 denoising (this section) was performed once per cohort using that cohort’s full read set, with no diagnosis labels or cross-cohort pooling, though not fully fold-isolated at the denoising stage for that reason. Zero-replacement/CLR (Section 2.3) is a per-sample deterministic function. Model tuning and fitting (Sections 2.4–2.5) use training-side samples only. The primary training-only genus-selection scheme closes the remaining pathway (feature selection informed by the test cohort’s own prevalence); the common-universe scheme does not, which is why it is not used as classifier-generalization evidence (Section 4.6).

### 2.3 Compositional data transformation

Raw genus-level count tables were converted to centered log-ratio (CLR) coordinates for all downstream analyses requiring distributional assumptions on unconstrained data (machine learning classifiers, PERMANOVA on Euclidean distance). The CLR transformation is defined as:

$$\mathrm{clr}(x_{i})=\log(\frac{x_{i}}{g(x)})$$

Where g(x) is the geometric mean of the composition x .

Zero counts were replaced prior to transformation using the multiplicative replacement strategy (Martín-Fernández et al., 2003) with δ=0.65 :

$$x_{i}^{*}=\{\begin{array}{ll} \delta & \text{if}\;x_{i}=0\\ (1-n_{0}\cdot \delta /T)\cdot x_{i} & \text{if}\;x_{i}>0 \end{array}$$

Where $T=\sum_{j}x_{j}$ is the total read count of the sample and $n_{0}$ is the count of zeros in the sample. The unified genus matrix contained 75.4% zero entries (151,926 of 201,564 cells across the 509 fecal samples and 396 genera). The value δ=0.65 was chosen such that the replacement mass remained small relative to typical per-sample sequencing depth (median 29,521 reads): in the worst-observed sample, the total mass allocated to zero-filled entries was 2.53% of that sample’s total reads. A built-in safeguard rescales δ downward whenever $n_{0}\cdot \delta \geq T$ , capping replacement mass at 1% of the sample total in such cases. This strategy preserves the ratios among non-zero components while introducing a minimal perturbation proportional to the relative frequency of zeros and is preferable to the commonly used additive pseudocount, which distorts low-abundance taxa more severely.

CLR-transformed values were used as features for all machine learning models and for Aitchison distance computation (Euclidean distance on CLR values). Raw genus-level count matrices (not CLR-transformed) were provided as input to ComBat-seq, which requires non-negative integer counts. Relative abundances (proportions) were provided to MMUPHin. Bray–Curtis dissimilarity was computed on relative abundances. CLR values were used for all other analyses.

### 2.4 Within-cohort classifier baselines

We trained binary classifiers (AD vs. CN) independently for each of the four labeled cohorts using two model families; this model-fitting methodology is shared by both feature-selection schemes compared in Section 2.5.1.

*L2-regularized logistic regression* (scikit-learn v1.5; Pedregosa et al., 2011; LogisticRegression, solver = “saga”). Regularization strength C was tuned over {0.001, 0.01, 0.1, 1.0, 10.0} in inner cross-validation. An l1_ratio parameter is present in the estimator configuration but has no effect under the L2 penalty actually used (scikit-learn’s penalty argument was not set to “elasticnet”); all logistic-regression results in this study are L2 (ridge)-regularized, not elastic-net.

*LightGBM gradient-boosted trees* (lightgbm v4; Ke et al., 2017). Hyperparameters tuned include the number of estimators ∈ {50, 100, 200}, learning rate ∈ {0.05, 0.1}, max depth ∈ {3, 5}, number of leaves ∈ {15, 31}.

For Li 2019, AD was defined as the positive class, and MCI samples were excluded from binary analyses (resulting in *n* = 60: 30 AD, 30 CN fecal samples); a separate three-class analysis (AD / MCI / CN) used all 90 fecal samples and is reported as a secondary finding. Because only fecal samples were retained, each patient appears exactly once; standard StratifiedKFold was used without any paired-sample grouping structure.

Performance was estimated using nested cross-validation: a 10-fold outer loop for performance estimation and a 5-fold inner loop for hyperparameter tuning. The primary metric was AUC-ROC computed on out-of-fold predictions pooled across the 10 outer folds, providing a single unbiased AUC estimate per cohort–model combination. Within-cohort 95% CIs (Table 1; Supplementary Figure S1) use the diagnosis-stratified, participant-level percentile bootstrap described in Section 2.5.2 (10,000 replicates); other CIs in this study use the non-stratified 1,000-resample bootstrap described in Section 2.5, as noted at each occurrence. We also report sensitivity and specificity at Youden’s J optimal threshold and note published AUC values from the original cohort studies in Table 1 where available.

#### 2.4.1 Li 2019 robustness: repeated cross-validation and label permutation

Because Li 2019’s near-perfect within-cohort AUC (Section 3.2) is estimated from only 60 samples (30 AD, 30 CN), we assessed its robustness with two additional analyses under the strict training-only feature-selection pipeline (Section 2.5.1), so that genus selection, model tuning, and model fitting used no held-out-fold information (DADA2 denoising, Section 2.2, is a per-cohort, label-free step performed once and is not re-derived per fold).

##### 2.4.1.1 Repeated nested cross-validation

The 10-fold outer / 5-fold inner nested-cross-validation (CV) procedure (Section 2.4) was repeated 50 times under different pre-specified deterministic seeds (1000–1,049) for the stratified outer-fold partition, with genus selection, zero-replacement, CLR transformation, and model tuning re-derived independently within every outer fold using only that fold’s training-side samples. We report the mean, SD, median, 2.5th/97.5th percentile, minimum, and maximum pooled-OOF AUC across the 50 repetitions. This quantifies sensitivity to fold assignment, not independent replication—the same 60 participants underlie all 50 repetitions.

##### 2.4.1.2 Label permutation test

Under the same pipeline and fixed outer-fold partition (random_state = 42): for each of exactly 1,000 permutations, AD/CN labels were shuffled across the 60 participants (preserving 30/30 class balance), and the classifier was refit on the permuted labels within every outer fold. Fold-specific genus selection, zero replacement, and CLR construction depend only on fold membership, not labels, so these were precomputed once and reused across permutations—an implementation optimization that does not alter the null, since the classifier itself is always refit on the permuted labels. Empirical p = (1+#{nullAUC≥observedAUC})/1001 ; the minimum attainable p at this permutation count is 1/1001≈0.000999 , not interpreted as more precise than that floor.

### 2.5 Cross-cohort generalization experiment

We evaluated two cross-cohort transfer designs:

*Leave-one-cohort-out (LOCO)*: For each test cohort, a classifier was trained on the pooled samples from the remaining three labeled cohorts (hyperparameters selected via 5-fold inner CV on the training pool) and then evaluated on the held-out cohort, producing four LOCO AUC estimates per model. The LOCO AUC drop is calculated as the within-cohort AUC minus the LOCO AUC, formally evaluated by the paired bootstrap method described in Section 2.5.2. Uncorrected LOCO 95% CIs (Table 2; Figure 2A) use the same diagnosis-stratified, participant-level bootstrap (10,000 replicates), while batch-corrected LOCO CIs (Table 3; Figure 3; Supplementary Figure S4) use the non-stratified 1,000-resample bootstrap described below.

- *Pairwise transfer*: A classifier was trained on one cohort and tested on each of the others individually, yielding a 4 × 4 matrix (excluding the diagonal) per model. This is more stringent than LOCO and isolates directional failures. Pairwise 95% CIs (Supplementary Table S1) use the non-stratified 1,000-resample bootstrap on test-set predictions.
- *Sensitivity analyses*: To assess whether generalization failure was attributable to any single cohort, we repeated LOCO excluding (A) Kazakhstan, (B) Zhuang 2018, and (C) Ling 2020 in turn (Section 3.3; Supplementary Figure S3).

**Table 3:** Mean LOCO AUC across four cohorts, by correction design and model (fixed common-universe schema throughout; not the Tables 1, 2 training-only primary pipeline).

Design	Method	LogReg mean LOCO AUC	LogReg mean drop from within-cohort	LGBM mean LOCO AUC	LGBM mean drop
—	Uncorrected	0.640	0.176	0.647	0.146
Label-blind (primary)	ComBat-seq	0.634	0.182	0.654	0.139
Label-blind (primary)	MMUPHin	0.639	0.176	0.648	0.145
Label-informed (exploratory)	ComBat-seq	0.493	0.322	0.555	0.238
Label-informed (exploratory)	MMUPHin	0.612	0.203	0.582	0.210

**Figure 3:**
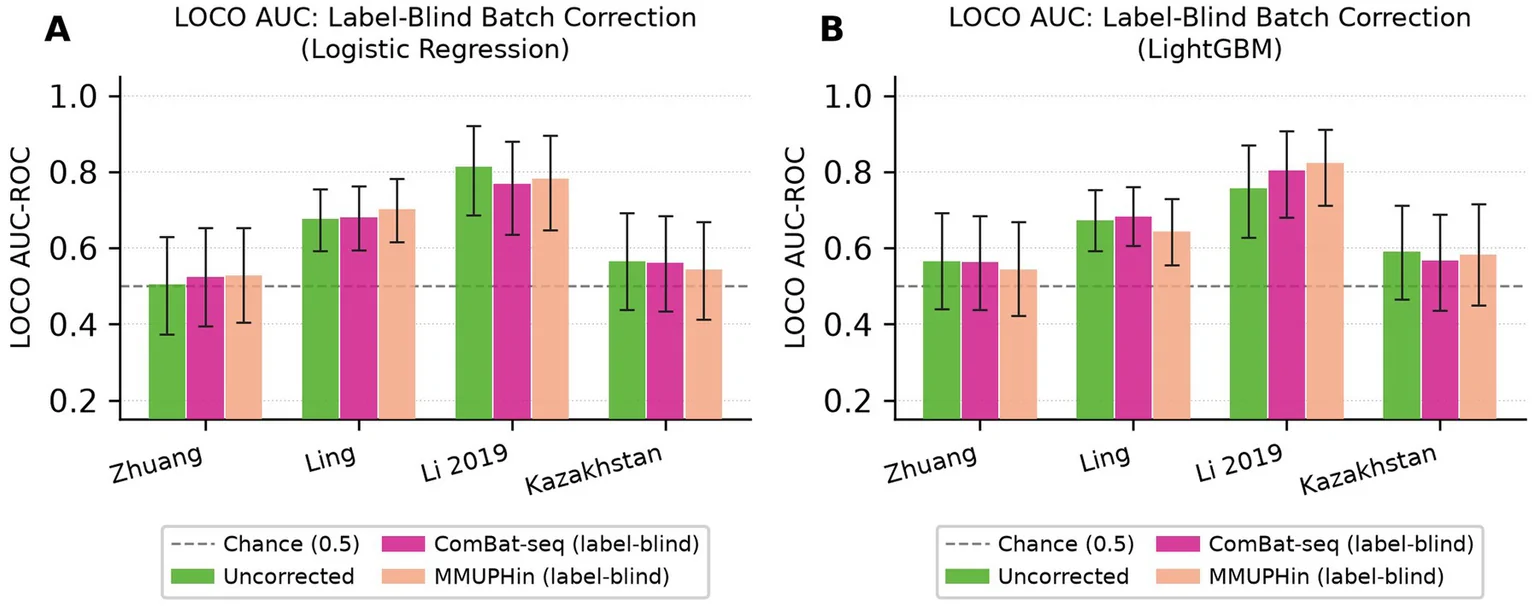
Effect of label-blind batch correction on LOCO cross-cohort AUC-ROC (primary analysis; no diagnosis information was used during correction). **(A)** Logistic regression and **(B)** LightGBM LOCO AUC for each held-out cohort under three conditions: uncorrected (green), ComBat-seq label-blind corrected (magenta), and MMUPHin label-blind corrected (salmon). Error bars represent the 95% bootstrap CI (1,000 non-stratified resamples on held-out test predictions; Section 2.5). Dashed line indicates chance (0.50). Under this label-blind design, both methods left mean LOCO AUC essentially unchanged relative to uncorrected (within 0.007 AUC for both methods and models), neither recovering nor substantially worsening cross-cohort generalization. An exploratory label-informed transductive sensitivity comparison, in which correction used each sample’s own true diagnosis label and produced substantially different, more severe degradation for some cohorts, is reported separately in Supplementary Figure S4 and is not treated as evidence of prospective performance (Section 2.7, Section 3.5).

#### 2.5.1 Primary feature selection: strict training-only versus the common-universe schema retained for secondary analyses

The global 396-genus set (Section 2.2) provided every LOCO/pairwise experiment with a feature universe partly informed by the held-out cohort’s own prevalence. To close this pathway, every headline within-cohort, LOCO, and pairwise-transfer AUC in this study (Tables 1, 2; Figures 1, 2A–C) instead uses genus selection re-derived independently within every split, from that split’s training-side samples only—this strict training-only scheme is the primary classifier-performance pipeline. For each outer fold/held-out cohort/train cohort, the ≥20%-prevalence filter was recomputed from training-side samples, and test/held-out samples were projected onto the training-derived genus set (with absent genera zero-filled) before zero-replacement and CLR. Model families, hyperparameter grids, random state, fold counts, and bootstrap procedures are otherwise identical to those in Sections 2.4–2.5.2. The retained genus count varies by split (e.g., 150 vs. 396 when Kazakhstan is excluded during its own LOCO hold-out); no headline classifier-generalization claim depends on held-out-cohort prevalence information.

The fixed common 396-genus universe is retained only for the secondary descriptive/harmonization analyses that require one common feature space across all cohorts simultaneously—such as batch correction (Section 2.7), SHAP/coefficient-stability/Jaccard-overlap (Section 2.8), and PERMANOVA (Section 2.6)—labeled as such at each occurrence and not used as classifier-generalization evidence. Both pipelines were computed side by side to confirm that the choice of the primary pipeline does not materially change conclusions (Section 3.3; Supplementary Tables S1, S4).

#### 2.5.2 Formal within-cohort vs. LOCO AUC difference

Non-overlapping bootstrap CIs are a heuristic, not a formal comparison. To formally evaluate the within-cohort versus LOCO AUC difference for each of the eight cohort–model combinations, we performed a paired, diagnosis-stratified, participant-level bootstrap: each of 10,000 replicates resampled participants with replacement (separately within AD and CN) and used that same resampled index set for both AUC values, so the resulting $\Delta ={\mathrm{AUC}}_{\text{within}}-{\mathrm{AUC}}_{\text{LOCO}}$ distribution is a genuine paired comparison. We report the observed delta, bootstrap mean delta, 95% percentile CI, and proportion of replicates with Δ≤0 . This same procedure regenerated the within-cohort and uncorrected-LOCO CIs in Tables 1, 2; pairwise-transfer CIs (Supplementary Table S1) and batch-corrected LOCO CIs (Table 3; Figure 3; Supplementary Figure S4) retain the original non-stratified 1,000-resample bootstrap of Section 2.5—each table/figure states which procedure its CIs use.

### 2.6 PERMANOVA variance decomposition

This section and Sections 2.7–2.8 use the fixed common 396-genus universe (Section 2.2), not the training-only primary pipeline, because each requires one common feature space across cohorts (Section 2.5.1). We conducted PERMANOVA using adonis2 (R package vegan v2.6; Oksanen et al., 2024) on Aitchison distance (Euclidean distance on CLR values, compositionally appropriate) and Bray–Curtis dissimilarity (relative abundances, for comparability with the broader literature).

All PERMANOVA models used 9,999 permutations with Type III (marginal) sums of squares (by = “margin”) for independent assessment of cohort and diagnosis effects; the minimum attainable permutation *p*-value is reported as *p* = 0.0001 throughout. Three models were fitted: (1) cohort only, all five cohorts (*n* = 509); (2) cohort + diagnosis, restricted to the four labeled cohorts (*n* = 401: AD and CN samples only, Li 2019; MCI excluded), with marginal *R*^2^ per term; (3) within each labeled cohort, diagnosis as the sole predictor (within-cohort disease effect size). Figure 4B displays the PCoA from Model 1 (*n* = 509, including MCI samples), while marginal R^2^ values reported throughout the text derive from Model 2 (*n* = 401).

**Figure 4:**
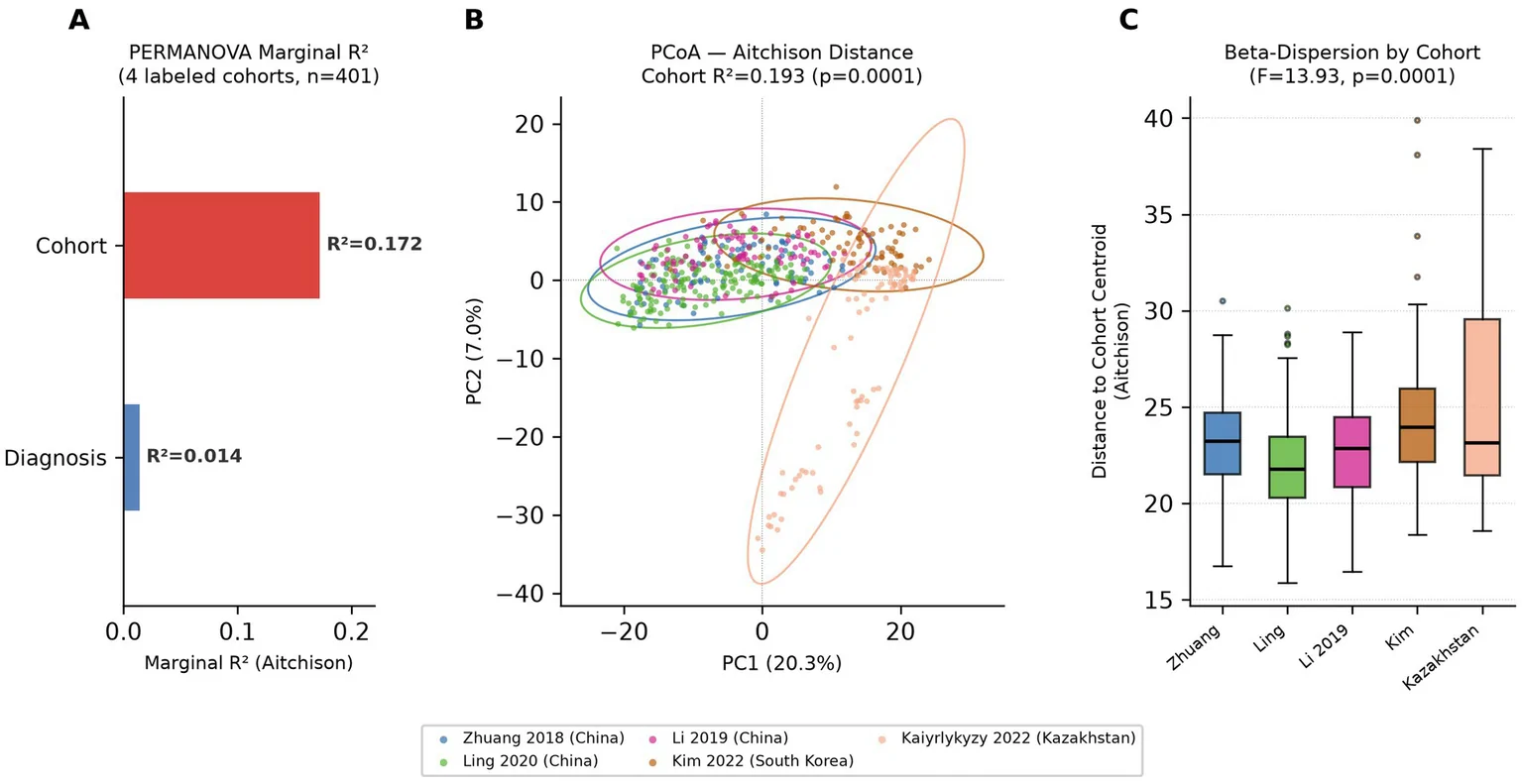
PERMANOVA variance decomposition and beta-diversity structure. **(A)** Marginal *R*^2^ values from a two-variable PERMANOVA model on the four labeled cohorts (*n* = 401): cohort *R*^2^ = 0.172 and diagnosis *R*^2^ = 0.014, shown as independent bars. These Type III marginal R^2^ values are estimated independently and do not sum to 1. **(B)** PCoA of the Aitchison distance matrix (all five cohorts; *n* = 509). Cohort *R*^2^ = 0.193 (*F* = 30.13, *p* = 0.0001, 9,999 permutations). Ellipses represent 95% normal confidence ellipses. **(C)** Beta-dispersion: distance from each sample to its cohort centroid (*F* = 13.93, *p* = 0.0001, 9,999 permutations).

Homogeneity of multivariate dispersions was tested using betadisper and permutest (9,999 permutations) to assess whether observed cohort differences reflected centroid separation, within-group spread heterogeneity, or both; a significant beta-dispersion test indicates that PERMANOVA *R*^2^ partially reflects within-group spread, not only centroid shifts. To identify which cohort pairs drive the overall dispersion difference, we additionally computed all pairwise betadisper comparisons among the five cohorts (Aitchison distance, 9,999 permutations per pair), with Holm and Benjamini–Hochberg multiplicity adjustment across the 10 pairwise *p*-values.

“Cohort” is a composite study-of-origin variable that PERMANOVA/betadisper cannot decompose into technical and biological sources (Section 4.6). To test how sensitive the cohort-versus-diagnosis disparity (Section 3.4) is to this, we repeated the same marginal two-variable PERMANOVA/betadisper specification (9,999 permutations, with pairwise post-hoc comparisons) on a subset of three more technically comparable cohorts—Zhuang 2018, Ling 2021, Li 2019, all Chinese paired-end MiSeq 2 × 300 bp V3–V4—restricted to binary AD/CN samples (*n* = 317).

To further quantify how much cohort-specific information is retained in the preprocessed feature space, we trained a logistic regression classifier (one-vs-rest, stratified 5-fold CV) to predict cohort identity from the CLR-transformed features across all 509 samples; the macro-AUC provides a complementary measure of cohort separability.

### 2.7 Batch correction

We evaluated one count-based batch-correction method (ComBat-seq) and one microbiome-specific meta-analysis framework (MMUPHin), each with cohort identity as the batch variable, under two designs differing in whether diagnosis information is supplied. Both are transductive: both estimate batch-effect parameters jointly across every corrected sample, including the eventual held-out test cohort’s own feature values and cohort membership in the downstream LOCO evaluation, and neither defines a transformation applicable to a genuinely new patient without refitting.

#### 2.7.1 Design 1—label-blind transductive correction (primary)

ComBat-seq (Zhang et al., 2020) was run on raw genus-level count matrices (genera × samples, four labeled cohorts) with group = NULL, full_mod = FALSE—no diagnosis information supplied. MMUPHin v1.15 (Ma et al., 2022) was run on relative-abundance profiles with batch = cohort, covariates = NULL; the metadata object given to adjust_batch included no diagnosis column. Both methods use cohort identity and every sample’s own raw feature values (making the design transductive) but never a true AD/CN label. Corrected tables were CLR-transformed using the zero-replacement procedure of Section 2.3.

#### 2.7.2 Design 2—label-informed transductive sensitivity analysis (exploratory)

The same methods were additionally run with group = disease_status, full_mod = TRUE (ComBat-seq) and covariates = diagnosis (MMUPHin), using every sample’s own true diagnosis label—including the eventual held-out cohort’s labels in the LOCO evaluation. Because correction is fit using test-cohort samples’ own diagnosis labels, this design cannot estimate prospective external-validation performance and is retained only as an exploratory sensitivity analysis, not as a performance bound: access to test labels during correction can improve or worsen downstream classifier performance, so it is not mathematically guaranteed to bound what label-blind correction could achieve.

After each correction, the LOCO experiment (Section 2.5) was repeated on the corresponding batch-corrected CLR matrices, and within-cohort AUC was re-computed under the same nested-CV procedure as in Section 2.4. Because Design 2’s correction step is explicitly given each sample’s own diagnosis label as part of a jointly fit model, within-cohort AUC on Design-2-corrected features cannot be used to argue that disease-associated biological signal survived correction independent of that label information—preserved separability could instead reflect the correction algorithm’s own label-preservation mechanism rather than biology. This caveat does not apply to Design 1, whose within-cohort AUC is not label-confounded, though it remains transductive like the LOCO evaluation itself.

### 2.8 SHAP feature importance, direction, and cross-cohort overlap analysis

To identify which genera drove predictions in each cohort and whether these features were consistent across cohorts, we computed SHapley Additive exPlanations (SHAP) values (Lundberg and Lee, 2017) using an out-of-fold (OOF) procedure to avoid the in-sample overfitting bias of computing SHAP on the same data used to fit the model.

For each labeled cohort, SHAP values were computed using the same 10-fold outer CV structure as AUC estimation (Section 2.4): in each fold, the classifier was trained on the 9 training folds, and SHAP values were computed for the held-out fold using the training fold as background. For logistic regression, shap. LinearExplainer was used with the training fold as background (feature_perturbation = “interventional”) and the held-out fold as the evaluation set, ensuring that the mean SHAP value is not algebraically forced to zero. For LightGBM, shap. TreeExplainer was used (check_additivity = False) with the same OOF structure. Values were aggregated across all 10 folds; mean absolute SHAP value per genus (|SHAP|) was used as the importance metric for both models and is unaffected by the direction issue below.

#### 2.8.1 Direction is not defined from mean signed SHAP

For a linear model under LinearExplainer with interventional perturbation, the SHAP value for feature *j* is approximately $\beta_{j}\cdot (x_{\mathrm{ij}}-{\overset{-}{x}}_{j}^{\text{background}})$ , so mean signed SHAP over an evaluation set is approximately $\beta_{j}\cdot ({\overset{-}{x}}_{j}^{\text{eval}}-{\overset{-}{x}}_{j}^{\text{background}})$ —depending on both the fitted coefficient and the mean-abundance difference between evaluation and background sets, not coefficient sign alone. Within a cohort’s own OOF folds, this difference is close to sampling noise, but for cross-cohort (LOCO) SHAP, the background (N − 1 training cohorts) and evaluation set (held-out cohort) come from systematically different populations by construction—the same compositional difference PERMANOVA quantifies in Section 2.6—so mean signed LOCO SHAP is confounded with raw cross-cohort abundance differences rather than reflecting the model’s direction. Mean signed SHAP is accordingly not used anywhere in this study as a global, cross-cohort direction or biological-association statistic.

#### 2.8.2 Coefficient-based direction and stability (logistic regression only)

For logistic regression, direction is defined from the fitted model’s own coefficient. In each of the 10 outer OOF folds per cohort, each genus’s coefficient sign was recorded. A genus’s direction in a cohort was stable—positive or negative—if the coefficient carried that sign in at least 8 of 10 outer folds (specified before any results were examined); an exactly zero coefficient counted toward neither. A genus met the descriptive coefficient-sign-stability screen only if it (a) appeared in the top-20 OOF mean-|SHAP| ranking in ≥2 cohorts, (b) had a stable positive coefficient in ≥1 of those cohorts, and (c) had a stable negative coefficient in ≥1 other—a conservative definition relative to a naive sign check on mean SHAP, since it requires the direction to be reproducible across most of the 10 outer folds, not merely to differ in one pooled mean-SHAP sign. We treat this screen as descriptive, not a formal hypothesis test: the 10 outer-CV folds within a cohort share substantially overlapping training data, so fold-level coefficient signs are not independent statistical replications, and no multiplicity-adjusted *p*-value is assigned to any taxon. LightGBM is a tree ensemble with no single coefficient-like, globally monotonic direction, and its SHAP-genus relationships may be non-linear; no direction or stability screen is therefore applied to LightGBM anywhere in this study—its results are limited to OOF mean-|SHAP| importance and the top-feature-overlap/Jaccard analysis below, neither of which requires a direction assignment.

For LOCO analyses, SHAP values were computed on the held-out test cohort from each LOCO-trained model, with the three-cohort training pool as background, and are used only as a feature *importance* ranking (mean |SHAP|); consistent with the confound above, no cross-cohort AD/CN direction is assigned to LOCO SHAP for either model.

#### 2.8.3 Cross-cohort taxonomic overlap

Pairwise taxonomic overlap was quantified using Jaccard similarity on the top-N SHAP genera (N ∈ {10, 20, 50}; N = 20 primary). The reported statistic is the mean of the six unique pairwise Jaccard values among the four labeled cohorts’ top-N sets ( $(\begin{array}{c} 4\\ 2 \end{array})=6$ ). Two null distributions are reported. The primary matched-statistic null mirrors the observed statistic’s construction exactly: in each of 100,000 replicates, four independent random top-N gene sets were drawn (without replacement) from the shared 396-genus pool, and the same six pairwise Jaccard similarities were averaged. Empirical one-sided p = $\left(1+\#\{\text{null}\geq \text{observed}\})/(n_{\text{perm}}+1\right)$ (Davison and Hinkley, 1997).

A second, prevalence-restricted null addresses a limitation of the shared pool: per-cohort genus prevalence varies substantially (e.g., Kazakhstan reaches ≥20% prevalence for 356/396 genera, versus 115–131 for the three MiSeq cohorts), so a genus nearly absent from a cohort could still be drawn into that cohort’s comparison set under the shared-pool null. Here, each cohort’s top-N ranking and random draws were both restricted to genera reaching ≥20% prevalence within that cohort’s own AD/CN-labeled samples, with 100,000 replicates per (model, N), and every cohort confirmed to have ≥N eligible genera. We report both nulls without assuming that significance under one implies significance under the other. Directional flips are classified using the coefficient-stability criterion above, not this overlap analysis.

### 2.9 AI tool disclosure

Initial manuscript drafts and computational scripts were prepared with minimal assistance from Claude Sonnet 5 (Anthropic). All analytical decisions, parameter choices, experimental design, statistical interpretations, and scientific conclusions were made by the author. All code and text were reviewed, validated, and substantially revised under the author’s direction.

### 2.10 Statistical analysis environment

DADA2 preprocessing (Section 2.2) was run using DADA2 v1.28 in R v4.3 and was not rerun for any analysis in this study. The initial classifier, PERMANOVA, batch-correction, and SHAP analyses (Sections 2.4–2.8, excluding the subsequent additions below) used scikit-learn v1.5, lightgbm v4, shap v0.51, pandas v2.x, and numpy v1.x (Python 3.11), along with vegan v2.6, sva v3.50 (ComBat-seq), and MMUPHin v1.15 in R v4.3. Subsequent robustness and sensitivity analyses (Sections 2.4.1, 2.5.1, 2.5.2, 2.6–2.8 sensitivity/robustness components) were executed in Python 3.11.15 (scikit-learn 1.5.2, lightgbm 4.6.0, shap 0.51.0, matching the original package versions) and R 4.5.1 (vegan 2.7.1, sva 3.58.0, MMUPHin 1.24.0, newer than the initial R environment, reflecting routine system updates); this mixed provenance is disclosed rather than described as uniform. Figures were generated with matplotlib v3.8 or v3.11 at 300 DPI.

### 2.11 Ethics statement

This study is a secondary analysis of publicly available, de-identified sequencing data and involved no direct participant recruitment, intervention, or access to identifiable information. Ethical approval and informed consent for primary data collection were the responsibility of the originating studies as reported in their publications; no new participants were recruited for the present analysis.

## 3 Results

### 3.1 Cohort characteristics and processing

Five cohorts comprising 599 deposited samples from three countries were assembled (Figure 5A; Table 4). After DADA2 processing and chimera removal, read retention rates varied substantially across cohorts (47.9–78.3%; Supplementary Table S2), reflecting differences in sequencing platform, library preparation, and amplicon length; the Kazakhstan cohort (single-end, NovaSeq) had notably different quality dynamics than the paired-end MiSeq cohorts. SILVA v138.1 genus-level classification and cross-cohort union produced a unified feature matrix of 396 genera. The 90 blood microbiome (B_*) samples from Li 2019 were excluded prior to all analyses (Section 2.4), leaving a working dataset of 509 samples across five cohorts. After construction of the global 396-genus feature schema, multiplicative zero replacement and CLR transformation were applied independently to each of the 509 samples, at δ = 0.65.

**Figure 5:**
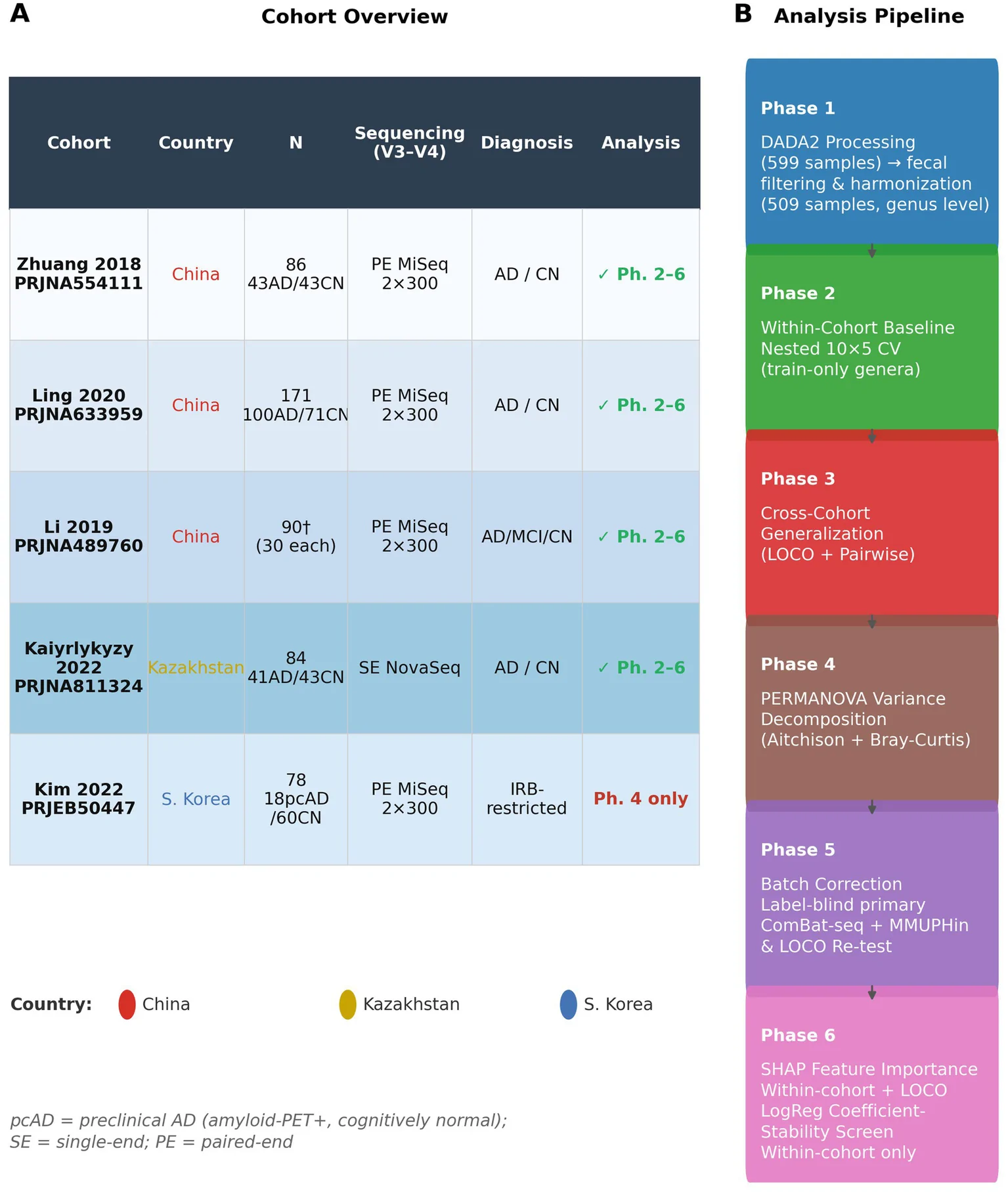
Study design. **(A)** Overview of the five 16S rRNA amplicon sequencing cohorts assembled for this study: four with per-sample diagnosis labels supporting supervised classification (Phases 2–3, 5–6) and a fifth (Kim/KBASE 2022) contributing only to unsupervised compositional analysis (Phase 4). Columns provide country of origin, sample size, sequencing mode, 16S variable region, diagnosis label availability, and analytic phases in which each cohort participates. **(B)** Summary of the six-phase analysis pipeline, from DADA2 processing through SHAP feature-importance and logistic-regression coefficient-stability analyses. Phases 2–3 (within-cohort baseline and cross-cohort generalization) use strict training-only genus selection, re-derived independently within every split, as the primary classifier-performance pipeline (Section 2.5.1); Phases 4–6 use the fixed common 396-genus universe (Section 2.2), which those specific analyses require as a single, shared feature space across cohorts.

**Table 4:** Cohort characteristics.

Cohort	Country	Accession	N (analyzed)	AD	CN	MCI	Sequencing	Library	Supervised analysis
Zhuang 2018	China	PRJNA554111	86	43	43	—	MiSeq 2 × 300	Paired-end	Phases 2–6
Ling 2020	China	PRJNA633959	171	100	71	—	MiSeq 2 × 300	Paired-end	Phases 2–6
Li 2019†	China	PRJNA489760	90 (fecal)	30	30	30	MiSeq 2 × 300	Paired-end	Phases 2–6
Kaiyrlykyzy 2022	Kazakhstan	PRJNA811324	84	41	43	—	NovaSeq	Single-end	Phases 2–6
Kim 2022	South Korea	PRJEB50447	78	18*	60	—	MiSeq 2 × 300	Paired-end	Phase 4 only
Total			509	232	247	30			

Amyloid-PET positive preclinical AD; per-sample labels are IRB-restricted and not publicly available. †Li 2019 deposited 180 samples (90 fecal F_ + 90 blood B_*); only fecal samples were retained for analysis.

For supervised classifier analyses (Phases 2–3 and 5–6), the Kim/KBASE 2022 cohort was excluded because per-sample diagnosis labels are IRB-restricted (Supplementary Note S1), leaving four labeled cohorts totaling 401 samples for binary AD/CN analyses (86 Zhuang + 171 Ling + 60 Zhu fecal binary subset + 84 Kazakhstan; Li 2019 MCI samples excluded).

### 3.2 Within-cohort classification performance

Within-cohort nested cross-validation, under strict training-only genus selection (primary analysis; Section 2.5.1), yielded AUC-ROC values broadly consistent with the original publications (Figure 1; Table 1). Logistic regression achieved an AUC-ROC of 0.665 [95% CI: 0.55–0.78] for Zhuang 2018, 0.890 [0.84–0.93] for Ling 2020, 0.996 [0.98–1.00] for Li 2019, and 0.737 [0.62–0.84] for Kazakhstan. LightGBM yielded similar values: 0.673 [0.56–0.78], 0.842 [0.78–0.90], 0.970 [0.93–1.00], and 0.611 [0.49–0.73] respectively. Matched common-universe values (Supplementary Table S4) agree closely (|Δ| ≤ 0.083 for every pair).

The near-perfect within-cohort AUC for Li 2019 fecal samples (logistic regression 0.996; LightGBM 0.970; *n* = 60 binary) is notable given the small sample size (30 per class): with 10-fold CV on 60 samples, each test fold contains ~6 samples, so fold-level AUC estimates can be unstable. PERMANOVA (Section 3.4) shows diagnosis explains *R*^2^ = 0.094 of within-cohort compositional variance (*F* = 6.01, *p* = 0.0001), indicating a statistically detectable diagnosis-associated difference in this cohort. A technical-metadata assessment of this cohort’s near-perfect AUC is reported in Section 4.3; the high AUC should be interpreted cautiously for that reason in addition to sample size, and the LOCO experiment (Section 3.3) provides a more informative assessment of transferability.

#### 3.2.1 Robustness to fold assignment and to label permutation (Section 2.4.1)

Repeating the training-only nested-CV partition 50 times under different pre-specified seeds gave a mean pooled-OOF AUC of 0.9955 (SD 0.0027, range 0.9889–0.9989) for logistic regression and 0.9760 (SD 0.0103, range 0.9378–0.9944) for LightGBM (Supplementary Table S8; Supplementary Figure S5), closely matching the single-partition primary values in Table 1 (0.996 and 0.970) and showing the near-perfect AUC is not an artifact of one fold assignment—a fold-assignment stability check, not independent replication, since the underlying 60 participants are identical across all 50 repetitions. A complementary label-permutation test under the same pipeline and fixed outer-fold partition (1,000 permutations) produced a null AUC distribution centered near chance for both models [logistic regression: mean 0.473, SD 0.102, 95% range (0.280, 0.673); LightGBM: mean 0.472, SD 0.109, 95% range (0.264, 0.688)]—well below the observed values (Supplementary Table S9; Supplementary Figure S5). No permutation among the 1,000 reached the observed AUC for either model, resulting in an empirical *p*-value at the resolution floor for this permutation count (*p* = 1/1,001 ≈ 0.000999 for both), which we do not interpret as more precise than this floor. Neither check distinguishes biological signal from a reproducible technical confound, which would pass both equally well (Section 4.3).

The three-class analysis (AD/MCI/CN) on all 90 Li 2019 fecal samples using logistic regression (one-vs-rest macro-AUC under nested cross-validation) yielded a macro-AUC of 0.761, confirming the multi-class discriminability of this cohort’s fecal compositional data.

For Zhuang 2018, within-cohort AUC values (0.665–0.673) were modest, consistent with the small sample size and weak within-cohort diagnostic effect observed in PERMANOVA (*R*^2^ = 0.017, *p* = 0.066; Section 3.4).

### 3.3 Cross-cohort generalization failure

LOCO cross-cohort AUC-ROC values, under strict training-only genus selection (primary analysis; Section 2.5.1), revealed substantial and variable degradation across all cohorts and model types (Figure 2A; Table 2). The mean LOCO AUC across the four cohorts was 0.652 for logistic regression and 0.641 for LightGBM—mean drops of 0.170 and 0.133, respectively, from within-cohort performance. One of the eight model–cohort combinations produced a LOCO AUC near chance: Zhuang 2018 with logistic regression [AUC = 0.506 (0.38–0.63)]; Kazakhstan LightGBM [AUC = 0.525 (0.40–0.65)] was modestly, but not substantially, above chance.

Under this primary pipeline, the largest absolute AUC drop for both models was for Ling (2020) (logistic regression 0.890 → 0.679, Δ = 0.210; LightGBM 0.842 → 0.653, Δ = 0.188). Li 2019 showed the next-largest drops for both models (logistic regression 0.996 → 0.812, Δ = 0.183; LightGBM 0.970 → 0.788, Δ = 0.182). These results are consistent with LOCO models relying on predictive patterns learned from training cohorts that do not transfer fully to the held-out cohort, where cohort-specific compositional structures may contribute to within-cohort performance. Kazakhstan and Zhuang 2018 showed smaller, though still substantial, drops (Table 2).

#### 3.3.1 Formal paired comparison of the within-cohort vs. LOCO AUC difference (Section 2.5.2)

A paired, diagnosis-stratified, participant-level bootstrap (10,000 replicates, identical resampled participants used for both AUC values in each replicate) applied to these training-only out-of-fold predictions formally compared the within-cohort and LOCO AUC differences for each of the eight cohort–model pairs, rather than relying on the non-overlap of independently computed CIs, which is not a formal test of a non-zero difference. Four of the eight pairs showed a 95% bootstrap CI on the delta excluding zero: Ling (2020) [logistic regression Δ = 0.210, 95% CI (0.12, 0.30); LightGBM Δ = 0.188, 95% CI (0.09, 0.28)] and Li 2019 [logistic regression Δ = 0.183, 95% CI (0.07, 0.31); LightGBM Δ = 0.182, 95% CI (0.07, 0.31)]. The remaining four—Zhuang 2018 logistic regression (Δ = 0.159, 95% CI [−0.02, 0.33], proportion Δ ≤ 0: 0.038), Zhuang 2018 LightGBM [Δ = 0.077, 95% CI (−0.10, 0.25), proportion Δ ≤ 0: 0.191], Kazakhstan logistic regression [Δ = 0.128, 95% CI (−0.03, 0.28), proportion Δ ≤ 0: 0.056], and Kazakhstan LightGBM [Δ = 0.086, 95% CI (−0.09, 0.27), proportion Δ ≤ 0: 0.179] — showed the same-signed (within-cohort higher) point estimate but a CI including zero, not formally significant despite the consistent direction across all eight pairs. The common-universe reference analysis yields five of the eight intervals excluding zero, whereas four of the eight do so under the primary training-only pipeline because the Kazakhstan logistic-regression interval includes zero under training-only selection. The aggregate pattern of reduced cross-cohort performance remains robust, but half of the eight individual comparisons do not reach formal statistical support at this sample size (full results in Supplementary Table S10).

LOCO performance was heterogeneous across cohorts: Li 2019 achieved the highest LOCO AUC (logistic regression 0.812), while Zhuang 2018 logistic regression and Kazakhstan LightGBM fell closest to chance. Six of eight main model–cohort combinations exceeded 0.55, so the results demonstrate substantial and variable degradation rather than uniform failure.

Sensitivity analyses confirmed that generalization failure was not attributable to Kazakhstan, Zhuang 2018, or Ling 2020 individually (Supplementary Figure S3; this cohort-exclusion check uses the fixed common-universe schema, Section 2.5.1, since it addresses cohort-composition robustness rather than feature-selection leakage). Excluding Kazakhstan, Zhuang 2018 remained near chance (LogReg 0.512, LightGBM 0.567); excluding Zhuang 2018, Kazakhstan LOCO remained substantially below its within-cohort baseline (LogReg 0.599, LightGBM 0.495); excluding Ling 2020, Kazakhstan LOCO LogReg was 0.604—confirming robustness across cohort compositions.

This pattern is not an artifact of the choice of pipeline: the common-universe reference values (Supplementary Table S4) differ by at most 0.066 for every within-cohort/LOCO pair, with no qualitative-classification change—the one material disagreement is a single pairwise-transfer cell, below.

Pairwise single-cohort transfer AUC under training-only selection (Figures 2B,C; Supplementary Table S1) ranged from 0.43 to 0.86. One cell changed materially and is reported without smoothing: Kazakhstan → Ling 2020 (LightGBM) fell from 0.58 (common universe) to 0.44 (training-only, Δ = −0.144), moving that cell from weak positive transfer to below chance—the largest shift in this comparison (full matrix and common-universe comparison: Supplementary Table S1). This does not change the overall heterogeneous/limited-transferability conclusion.

### 3.4 PERMANOVA variance decomposition

PERMANOVA on Aitchison distance across all five cohorts showed cohort identity explained 19.3% of total compositional variance (*R*^2^ = 0.193, *F* = 30.13, *p* = 0.0001, 9,999 permutations; Figure 4B). On Bray–Curtis dissimilarity, cohort *R*^2^ was 0.136 (*F* = 19.80, *p* = 0.0001). Cohort-associated compositional variation was substantially larger than diagnosis-associated variation in this pooled four-cohort model: in a marginal two-variable model restricted to the four labeled cohorts (*n* = 401), cohort explained *R*^2^ = 0.172 (*F* = 28.03, *p* = 0.0001) versus diagnosis *R*^2^ = 0.014 (*F* = 6.82, *p* = 0.0001)—an approximately 12-fold disparity within the same model. This is an empirical statement about relative magnitude, not a decomposition of cohort into technical and biological components: “cohort” bundles sequencing platform, library preparation, recruitment site, diagnostic ascertainment, and unmeasured dietary/lifestyle factors that PERMANOVA cannot separate (Section 2.6). It contextualizes the batch-correction results in Section 3.5, but marginal R^2^ values do not by themselves establish that the cohort and diagnosis axes overlap, nor explain why any batch-correction algorithm changed classifier performance in a specific direction—discussed as a separate, more limited hypothesis in Section 4.2.

*The ~12-fold disparity is sensitive to cohort composition and should not be read as a fixed, generalizable figure*. Restricting the same marginal specification to the three more technically comparable cohorts—Zhuang 2018, Ling 2020, and Li 2019, all Chinese, paired-end MiSeq V3–V4 studies (Section 2.6)—reduced the disparity from ~12-fold to ~2.6-fold: cohort *R*^2^ = 0.0588 (*F* = 10.05, *p* = 0.0001) versus diagnosis *R*^2^ = 0.0229 (*F* = 7.83, *p* = 0.0001) in this *n* = 317 subset (Supplementary Table S11). This subset is more comparable, not identical—recruitment site, exact diagnostic procedure, and unmeasured dietary factors still differ—but removing Kazakhstan’s single-end/NovaSeq platform and Kim/KBASE’s separate country substantially changes the cohort-to-diagnosis variance ratio. The ~12-fold figure for the full four-cohort set is therefore not a stable, generalizable property of “cohort effects versus disease effects” in gut microbiome data; it is specific to the cohorts compared and is driven substantially by cohorts differing in more than just country of origin.

A logistic regression classifier trained to predict cohort identity from the CLR-transformed features across all 509 samples achieved macro-AUC = 0.9917 (one-vs-rest, stratified 5-fold CV; per-cohort AUC: Kazakhstan 1.000, Kim 2022 0.997, Ling 2020 0.993, Zhuang 2018 0.989, Li 2019 0.980)—cohort membership is thus nearly perfectly recoverable from the same features used for disease classification, confirming that cohort-specific information (regardless of its technical or biological composition) dominates the compositional feature space.

Principal coordinates analysis (PCoA) on Aitchison distance (Figure 4B; n = 509) visually confirmed strong cohort-level clustering, with partial overlap between the Chinese cohorts (Zhuang 2018, Ling 2020) but clear separation of Kazakhstan and Li 2019. Kim/KBASE 2022 was clearly separated from all others along PC1, consistent with its preclinical amyloid-based staging, South Korean origin, and potentially distinct dietary patterns.

Beta-dispersion testing revealed significant heterogeneity in within-group spread across cohorts (*F* = 13.93, *p* = 0.0001, 9,999 permutations; Figure 4C), meaning PERMANOVA R^2^ = 0.193 reflects both compositional centroid shifts and differences in within-cohort spread. Kazakhstan showed the greatest within-cohort variance, while the Chinese cohorts (Zhuang 2018, Ling 2020) were more tightly dispersed. Pairwise post-hoc betadisper comparisons (Section 2.6; Supplementary Table S12) show this is not uniform across all pairs: Ling 2020 differs significantly from every other cohort (all Holm-adjusted *p* ≤ 0.0420) and drives most of the significant comparisons, while Li 2019 vs. Zhuang 2018 (Holm *p* = 0.439) and Kazakhstan vs. Kim/KBASE 2022 (Holm *p* = 0.439) do not differ significantly. Within the Chinese-MiSeq-only subset, dispersion remained significant overall (*F* = 7.946, *p* = 0.0004): Zhuang 2018 differed significantly from Ling 2020 after Holm correction (raw *p* = 0.0001, Holm-adjusted *p* = 0.0003), but its difference from Li 2019 did not survive Holm correction (raw *p* = 0.026, Holm-adjusted *p* = 0.052), and Ling 2020 and Li 2019 did not differ (*p* = 0.343). This heterogeneity is relevant to interpreting PERMANOVA effect sizes and is acknowledged in Methods and Limitations.

Within-cohort PERMANOVA revealed highly variable disease-associated compositional effects: Ling 2020 (*R*^2^ = 0.053, *F* = 9.53, *p* = 0.0001) and Li 2019 (*R*^2^ = 0.094, *F* = 6.01, *p* = 0.0001) showed significant diagnostic effects, while Zhuang 2018 (*R*^2^ = 0.017, *p* = 0.066) and Kazakhstan (*R*^2^ = 0.022, *p* = 0.069) did not reach significance at *α* = 0.05. These effect sizes broadly rank-order with within-cohort AUC performance, triangulating the classifier results. Li 2019’s fecal-only *R*^2^ = 0.094 is the highest within-cohort disease effect among the four labeled cohorts, consistent with a detectable diagnosis-associated compositional difference.

### 3.5 Label-blind transductive batch adjustment does not improve cross-cohort transferability

Batch correction was applied using ComBat-seq (raw counts) and MMUPHin (relative abundances) to the four labeled cohorts with cohort as the batch variable, under the two designs of Section 2.7: a label-blind design (no diagnosis information used; primary analysis) and a label-informed design (each sample’s own true diagnosis label used; exploratory sensitivity only; Figure 3; Table 3). This entire experiment, including its uncorrected baseline, uses the fixed common 396-genus universe (Section 2.2), not the training-only pipeline primarily referenced in Tables 1, 2 (Section 2.5.1), since ComBat-seq/MMUPHin require one shared feature matrix—so Table 3’s “Uncorrected” row is this experiment’s own matched baseline, not the Tables 1, 2 numbers, keeping uncorrected-vs-corrected comparisons apples-to-apples within this experiment.

*Label-blind correction (primary result): batch correction leaves cross-cohort generalization essentially unchanged*. Under label-blind ComBat-seq and MMUPHin—which use no sample’s diagnosis label—mean LOCO AUC changed by no more than 0.007 in either direction relative to uncorrected, for either model (Table 3). No individual cohort collapsed: per-cohort logistic-regression LOCO AUC under label-blind ComBat-seq ranged from 0.525 (Zhuang 2018) to 0.769 (Li 2019), closely tracking the uncorrected values (0.504–0.813). Neither method materially changed mean LOCO AUC or recovered cross-cohort transferability under this design.

*Label-informed transductive sensitivity analysis (**Supplementary Figure S4**): severe degradation was specific to the label-informed design itself*. Under the label-informed design—which uses every sample’s own true diagnosis label during correction and therefore cannot represent a deployable or prospectively valid comparison—ComBat-seq reduced mean logistic-regression LOCO AUC from 0.640 to 0.493, with Kazakhstan collapsing to AUC = 0.308 [0.20–0.42]. The same cohort under label-blind ComBat-seq scored 0.560, closely matching its uncorrected value (0.564). This side-by-side comparison indicates that the severe, cohort-specific collapse was specific to the label-informed design—consistent with a correction model explicitly informed of each sample’s diagnosis label (full_mod = TRUE/covariates = “diagnosis”) constructing a label-conditional transformation with no prospective analog—and should not be interpreted as evidence that these methods generally degrade cross-cohort performance or indicate an intrinsic incompatibility between ComBat-seq’s negative-binomial framework and 16S compositional data. MMUPHin’s label-informed effect was milder (LogReg mean: 0.640 → 0.612) but still did not improve on label-blind or uncorrected performance.

Within-cohort AUC on label-blind-corrected data was similar to uncorrected (within ±0.05 for most cohort–method combinations; Supplementary Table S5)—a comparison free of diagnosis-label leakage during correction since label-blind correction never saw any sample’s diagnosis label. However, it remains transductive (cohort membership and raw feature values are still used) and retains whatever cohort/population confounding is otherwise present. We do not draw the same conclusion from within-cohort AUC on label-informed-corrected data, since that comparison is additionally confounded by label use: the label-informed correction’s covariate-preservation mechanism (full_mod = TRUE/covariates = “diagnosis”) is explicitly designed to retain diagnosis-associated structure using each sample’s true label, so preserved within-cohort separability cannot be attributed to correction-independent biological signal versus the algorithm’s own label-preservation step. Only the label-blind design supports the conclusion that “within-cohort AUC preservation rules out indiscriminate signal removal” (descriptively, per Supplementary Table S5); we do not extend this conclusion to the label-informed results.

The empirical PERMANOVA finding that cohort explains approximately 12 times more compositional variance than disease status (marginal *R*^2^: 0.172 vs. 0.014; Section 3.4) contextualizes why a correction method targeting cohort-associated variance might struggle to leave disease-associated variance fully intact but does not by itself establish that the two sources overlap, nor explain the specific pattern observed here—a label-blind correction leaving performance essentially flat while a label-informed one produced large, method- and cohort-specific movements. That pattern is more directly explained by the transductive, label-informed design itself (Section 2.7) than by the general cohort/diagnosis variance disparity, and we present it as such rather than as a demonstration that linear batch correction cannot separate cohort from disease signal in general.

### 3.6 SHAP analysis reveals cohort-specific taxonomic signatures and a descriptive coefficient-stability screen

To characterize feature-level patterns underlying generalization failure, we computed out-of-fold SHAP values for within-cohort and LOCO-trained classifiers (Figure 6). The OOF design avoids the in-sample overfitting bias seen in prior SHAP analyses of microbiome classifiers, where training-set SHAP values can reflect model memorization rather than out-of-sample importance. Feature importance (mean |SHAP|) is reported for both models; direction is reported only for logistic regression, using the fitted-coefficient-based definition of Section 2.8 (not mean signed SHAP).

**Figure 6:**
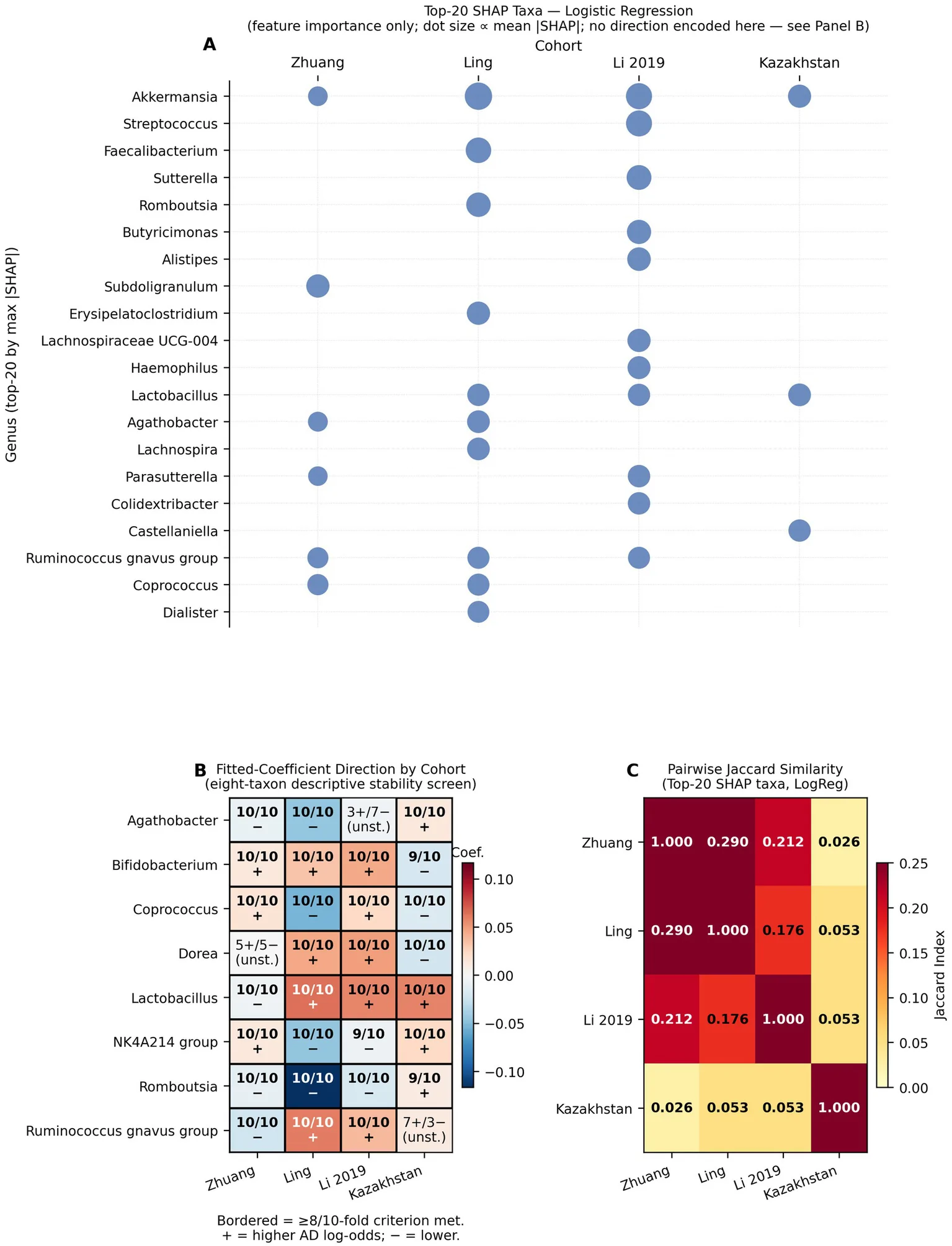
SHAP feature importance and coefficient-based direction analysis, logistic regression. **(A)** Top-20 SHAP genera by maximum |SHAP| across cohorts. Dot size ∝ mean |SHAP|; feature importance only—no direction is encoded in this panel. **(B)** Fitted-coefficient direction by cohort for the eight genera meeting a pre-specified descriptive coefficient-sign-stability screen (top-20 in ≥2 cohorts; a stable positive coefficient sign in ≥8/10 outer folds in ≥1 cohort and a stable negative sign in ≥8/10 folds in ≥1 other). Cell color = median fitted logistic-regression coefficient across the 10 outer folds; cell text = fold count and direction. Bordered cells meet the ≥8/10 stability criterion. This is a descriptive screen, not a multiplicity-corrected hypothesis test. **(C)** Pairwise Jaccard similarity of top-20 SHAP taxa between cohort pairs; the mean of the six unique pairwise values = 0.135, significantly above a null matched to this statistic’s construction (100,000 replicates of four random top-20 sets; *p* < 0.0001). A prevalence-restricted null sensitivity analysis (Supplementary Table S13) shows logistic regression remains significant, while LightGBM does not.

*Minimal overlap in top predictive taxa across cohorts, assessed against two null models*. Pairwise Jaccard similarity of the top-20 SHAP genera between cohort pairs averaged 0.135 for logistic regression and 0.058 for LightGBM (Figure 6C)—the mean of the six unique pairwise Jaccard values among the four labeled cohorts. Against the primary shared-pool null (100,000 replicates, each averaging six pairwise Jaccards among four random top-20 sets drawn from the full 396-genus pool, Section 2.8), logistic regression overlap was significantly above chance (null mean 0.027 [95% CI, 0.009–0.049]; *p* < 0.0001), as was LightGBM at this N (*p* = 0.0048). A sensitivity check across N∈{10,20,50} under this null (Supplementary Table S7) shows logistic regression significant at all three thresholds, while LightGBM was significant at N = 20 and *N* = 50 but not N = 10 (*p* = 0.45).

*Under a more realistic, per-cohort prevalence-restricted null, the LightGBM finding does not survive*. The shared-pool null draws candidate genera as if they are equally available in every cohort, but per-cohort prevalence varies substantially (Section 2.8). Restricting each cohort’s top-N ranking and random draws to genera reaching ≥20% prevalence within that cohort’s own AD/CN samples (eligible universe: 131 Zhuang 2018, 126 Ling 2020, 115 Li 2019, 356 Kazakhstan; 100,000 replicates per model/N) changes the LightGBM result: overlap was not significant at *N* = 10, 20, or 50 (observed mean Jaccard 0.018/0.063/0.167 vs. null 0.026/0.052/0.142; *p* = 0.80, 0.24, 0.061; Supplementary Table S13). Logistic regression remained significant at all three thresholds (observed 0.085/0.135/0.231 vs. null 0.026/0.052/0.142; *p* = 0.0017, 0.00003, 0.00001). We treat this as a genuine reversal: LightGBM’s apparent cross-cohort overlap is not distinguishable from chance once the null accounts for each cohort’s own prevalence structure, while logistic regression’s overlap is robust and remains the stronger of the two findings.

*Cohort-specific top predictors and a concrete illustration of why coefficient-based direction was needed*. The most discriminative genus for logistic regression was *Subdoligranulum* in Zhuang 2018, while *Akkermansia* topped the remaining three cohorts (Li 2019 |SHAP| = 0.228; Kazakhstan |SHAP| = 0.136; Ling 2020 |SHAP| = 0.270). Under the fitted coefficient, *Akkermansia* has a stable positive coefficient in all four cohorts (10/10 outer folds positive in every cohort; Supplementary Table S6)—higher CLR abundance consistently predicts higher AD log-odds, with no coefficient-based flip anywhere. This is a useful negative control: pooled mean SHAP for this genus is negative in Ling 2020 and Zhuang 2018 but positive in Li 2019 and Kazakhstan—an apparent sign difference that does **not** reflect the coefficient actually learned, but is instead a background-relative artifact (Section 2.8), demonstrating on real data why mean signed SHAP was inadequate and why the coefficient-based definition is used for every direction claim in this study. For LightGBM, the top taxon per cohort was entirely distinct: *Lachnoclostridium* (Zhuang 2018), *Akkermansia* (Ling 2020), *Bacteroides* (Li 2019), *Castellaniella* (Kazakhstan) (Figure 6A; Supplementary Figure S2); no direction is assigned to these values (Section 2.8).

*Coefficient-sign stability screen (logistic regression only)*. Using the pre-specified criterion (Section 2.8: stable positive coefficient sign in ≥8/10 outer folds in ≥1 cohort, stable negative in ≥8/10 folds in ≥1 other, restricted to genera in the top-20 OOF mean-|SHAP| ranking in ≥2 cohorts), eight genera met this descriptive stability criterion (Figure 6B): *Agathobacter*, *Bifidobacterium*, *Coprococcus*, *Dorea*, *Lactobacillus*, *NK4A214 group*, *Romboutsia*, and *Ruminococcus gnavus group*. This is deliberately a descriptive screen, not a statistically confirmed set: the 10 outer-CV folds share substantially overlapping training sets, so fold-level coefficient signs are not independent replications, and no multiplicity-adjusted *p*-value is assigned to any taxon (Section 2.8, Limitations). It is a smaller, more conservative set than a less rigorous alternative that classifies any genus with opposite-signed pooled mean SHAP across two cohorts as a “flip”—which, applied here, would identify 12 genera; the stability requirement removes flips not reproducible across most of a cohort’s own outer folds. No comparable screen is applied to LightGBM, which has no coefficient-like, globally monotonic direction and may have non-linear SHAP–feature relationships.

*Romboutsia* shows the cleanest cohort-level pattern among the eight taxa meeting the screen: a stable negative coefficient (higher relative abundance predicts lower AD log-odds under the fitted model) in all three Chinese cohorts and a stable positive coefficient in Kazakhstan (9/10 folds; Supplementary Table S6)—the one genus among the eight whose pattern cleanly separates by country in this dataset. We do not have a specific mechanistic hypothesis for *Romboutsia*.

*Lactobacillus* also meets the screen, but its per-cohort pattern is messier: stable negative in Zhuang 2018 only, and stable positive in the other three cohorts (Ling 2020, Li 2019, Kazakhstan; Supplementary Table S6). Diet is one plausible unmeasured confounder for genus-level differences of this kind, but this dataset contains no dietary covariate data, and we do not attribute this or any other taxon’s pattern to a specific mechanism.

*Akkermansia* does not meet the coefficient-stability screen at all: its fitted coefficient is stable and positive in all four cohorts (10/10 outer folds in every cohort; Supplementary Table S6), despite appearing to reverse sign under the pooled mean-SHAP statistic (above). This is the clearest illustration in this dataset of why coefficient-based, fold-stability-gated direction is necessary rather than a raw pooled-SHAP sign.

*LOCO SHAP analysis: feature importance only, no cross-cohort direction assigned*. When SHAP values were computed for LOCO-trained classifiers applied to held-out cohorts, *Akkermansia* was the top-importance predictor for two of the four held-out cohorts—Zhuang 2018 (|SHAP| = 0.470) and Li 2019 (|SHAP| = 0.372)—while *Subdoligranulum* (|SHAP| = 0.269) topped held-out Ling 2020 and the *Christensenellaceae R-7 group* (|SHAP| = 0.368) topped held-out Kazakhstan; no genus ranked first across all four. Consistent with Section 2.8, we do not assign an AD/CN direction to these values: the LOCO SHAP background (three training cohorts) and evaluation set (held-out cohort) are drawn from systematically different populations by construction, confounding sign with the raw cross-cohort compositional difference quantified by PERMANOVA (Section 3.4) rather than reflecting a stable, model-derived direction. The absence of a single top-importance genus across all four held-out cohorts is consistent with the classifier activating whichever genera most resemble its training-cohort signature rather than a disease marker generalizing across populations.

## 4 Discussion

### 4.1 Systematic quantification of generalization failure

This study provides a systematic, quantitative analysis of cross-cohort generalization limitations in gut microbiome-based AD classifiers. Across four labeled cohorts, two model types, two cross-cohort evaluation designs, and two batch-correction methods, we consistently observe the same result: classifiers trained within one cohort do not transfer reliably to another. The mean LOCO AUC drop of 0.170 for logistic regression and 0.133 for LightGBM (strict training-only pipeline, primary; Section 2.5.1), with one of eight model–cohort combinations near chance, demonstrates that within-cohort performance figures—from the original publications or from this study’s own nested-CV estimates—cannot be taken as evidence of a generalizable diagnostic signal. A formal paired bootstrap test (Section 3.3) supports this drop for four of the eight cohort–model pairs at conventional significance; the remaining four show the same directional pattern without reaching formal significance at this sample size, a distinction we report rather than treat all eight as equally well-supported.

This finding is consistent with the broader literature. The *Bacteroides* directionality reversal documented by Jemimah et al. (2023) across North American and Chinese cohorts, contradictory reports for *Akkermansia* (Liu et al., 2019), and highly cohort-specific patterns reported elsewhere all suggest population-level heterogeneity. A recent multi-cohort integration study (Wang et al., 2026, mSystems) similarly identified cross-geographic heterogeneity as a major obstacle; the present analysis complements that work by quantifying how severely within-cohort classifiers fail on external populations and by showing that neither correction method recovered cross-cohort transferability on average.

### 4.2 Batch-adjustment results and limits of mechanistic interpretation

Two distinct findings must be kept separate here.

#### 4.2.1 Empirical result

Cohort identity explains substantially more compositional variance than disease status (Aitchison *R*^2^ = 0.193 across all five cohorts; marginal *R*^2^ = 0.172 for cohort vs. 0.014 for diagnosis in the four-cohort two-variable model—an approximately 12-fold disparity), and cohort membership is near-perfectly recoverable from the same CLR features (macro-AUC = 0.9917). This 12-fold figure is not fixed, however: restricted to the three more technically comparable Chinese paired-end MiSeq cohorts, the same comparison decreases to approximately 2.6-fold (cohort *R*^2^ = 0.059 vs. diagnosis *R*^2^ = 0.023; Section 3.4), and the significant beta-dispersion difference among cohorts (Section 3.4) means these *R*^2^ values reflect both centroid separation and within-cohort spread, limiting a simple centroid-distance interpretation of cohort separation. Compositional structure was strongly associated with cohort of origin in the evaluated datasets, but the magnitude is sensitive to cohort composition and dispersion structure, not a single stable number. Separately, batch correction, evaluated under a label-blind design using no sample’s diagnosis label, left mean LOCO AUC essentially unchanged (within 0.007 AUC of uncorrected for both methods and both models; Section 3.5)—neither recovering cross-cohort transferability nor substantially degrading it.

#### 4.2.2 Hypothesis, clearly flagged as such

The much larger cohort-associated variance component provides plausible context for *why* correction targeting cohort-associated variance might struggle to isolate and remove only that variance without also touching disease-associated variance if the two are not orthogonal in this compositional space. PERMANOVA marginal R^2^ values do not establish that overlap directly, and we did not run an experiment (e.g., a simulation with known, controlled cohort/diagnosis-axis overlap) capable of testing it. We present this as a hypothesis consistent with the observed pattern, not a demonstrated mechanism, and no longer state that batch correction cannot work in general or that this analysis proves cohort and disease axes overlap.

Under the label-informed design, in which ComBat-seq and MMUPHin were told each sample’s true diagnosis label during a joint fit across all four labeled cohorts (Section 2.7), ComBat-seq’s correction substantially worsened LOCO AUC (mean LogReg: 0.640 → 0.493, one cohort collapsing to AUC = 0.308). The identical comparison under a label-blind design—same cohorts, features, and downstream classifiers, only omitting the diagnosis label from correction—instead reproduces essentially flat LOCO AUC (Section 3.5, Table 3), indicating the severe degradation was specific to the label-informed design rather than evidence that these methods generally degrade cross-cohort performance or indicate an intrinsic incompatibility between ComBat-seq’s negative-binomial framework and 16S compositional data. One hypothesis consistent with this pattern, though not established by it, is that a correction model explicitly told each sample’s diagnosis label constructs a label-conditional transformation with no prospective analog. We therefore do not attribute the label-informed collapse to a property of ComBat-seq’s modeling framework and retain those numbers only as an explicitly labeled exploratory sensitivity illustrating what a label-aware, non-deployable correction can do (Section 3.5).

The finding that survives this correction—and that we consider the more robust and useful result for the field—is narrower than originally stated: under label-blind transductive evaluation, two widely used batch-correction methods designed for other data types (RNA-seq counts; general microbiome meta-analysis) did not recover cross-cohort transferability for AD classification in this dataset. This demonstrates ineffectiveness at the specific task, not that these methods actively destroy signal, nor a mechanistic explanation of why such correction is difficult. The field may still need fundamentally different approaches—multi-cohort training with cohort as an explicit stratification variable, domain adaptation methods, or meta-analytic frameworks that model between-cohort heterogeneity rather than attempting to remove it—but we no longer claim to have shown mechanistically why standard correction fails, only that it did not succeed under the conditions tested here.

### 4.3 Implications of the Li 2019 paired-sample design

The Li 2019 cohort deposited both blood microbiome (B_) and fecal (F_) samples from the same 90 participants (PRJNA489760). Blood samples were excluded because blood and fecal microbiome communities are fundamentally distinct compartments; including both would introduce a sample-type confound structurally identical to a batch effect, inflating within-cohort AUC through tissue-compartment discrimination rather than disease discrimination.

With fecal-only samples (n = 60 binary), the near-perfect within-cohort AUC (logistic regression 0.996, LightGBM 0.970; primary training-only pipeline, Table 1) is harder to attribute to a single confound. The small sample size (30 per class) is relevant—each 10-fold CV test fold contains ~6 samples, so single-fold AUC estimates can be highly variable—and Li 2019 showed the largest detectable diagnosis-associated compositional effect (R^2^ = 0.094, *F* = 6.01, *p* = 0.0001) of the four labeled cohorts. The repeated-CV and label-permutation analyses (Section 2.4.1, Section 3.2) show this estimate is stable to fold assignment and clearly distinguishable from a chance-label null; it may nonetheless overstate population-level performance, being derived from only 60 participants without independent replication.

#### 4.3.1 Technical-metadata assessment of sequencing-batch/diagnosis confounding

To assess potential sequencing-batch/diagnosis confounding in Li 2019, we examined this cohort’s SRA technical metadata (full field-by-field results: Supplementary Table S14; Supplementary Note S4). Mean read length (avgLength) is exactly associated with diagnosis (all 30 AD runs = 600 bp, all 30 CN runs = 602 bp); other technical fields (release timestamp, accession order) show a concordant but non-exact pattern, and no lane/plate/extraction-batch/run identifier has been deposited, so a physical batch cannot be identified directly. Our DADA2 pipeline truncates all reads in this cohort to one fixed length regardless of raw read length, so this 2-bp difference cannot itself survive into the genus table as a length artifact—but its exact collinearity with diagnosis is consistent with a correlated technical grouping whose other consequences could still affect composition estimates. Because diagnosis and avgLength are exactly collinear, no adjustment can separate a technical from a biological explanation, and we do not attempt to; we report this as a disclosed, unresolved limitation, not as a demonstrated artifact. Critically, the repeated-CV and label-permutation results above establish fold stability and non-chance significance, not a biological origin—a reproducible technical confound would pass both checks equally well.

The LOCO result for Li 2019 (logistic regression AUC = 0.812, the highest LOCO value among all cohorts) is informative: classifiers trained on the other three cohorts transfer to Li 2019 fecal samples better than to any other cohort, suggesting the classification pattern learned here is somewhat consistent with the Chinese and Kazakh training cohorts, unlike a spurious compartment-discrimination signal that mixed-compartment pooling would have produced. This LOCO result does not, however, resolve the within-cohort confounding question above, since it concerns classifiers trained on other cohorts. We recommend that future AD microbiome studies report sample types explicitly and deposit lane/plate/extraction-batch metadata to permit exactly this kind of check.

### 4.4 Coefficient-sign stability screen: descriptive cross-cohort direction heterogeneity

As reported in Section 3.6, eight genera met the pre-specified coefficient-sign-stability screen (≥8/10 outer folds agreeing in sign; Section 2.8) for logistic regression: *Agathobacter*, *Bifidobacterium*, *Coprococcus*, *Dorea*, *Lactobacillus*, *NK4A214 group*, *Romboutsia*, and *Ruminococcus gnavus group*. We present this descriptively, not as statistically confirmed contradictions—the 10 outer-CV folds share overlapping training data, so fold-level signs are not independent replications, and we assign no multiplicity-adjusted *p*-values. A less conservative alternative (opposite-signed pooled mean SHAP across two cohorts, a background-relative quantity, not the coefficient) would identify 12 genera for each model; requiring reproducibility across a cohort’s own outer folds and grounding direction in the fitted coefficient is why this study reports eight genera for logistic regression and none for LightGBM, which has no coefficient-like global direction to test.

*Romboutsia* shows the cleanest cohort-level pattern: stable-negative in all three Chinese cohorts and stable-positive in Kazakhstan; we offer no mechanistic hypothesis. *Lactobacillus* also meets the screen but does not cleanly separate by country (stable-negative in Zhuang 2018 only). Diet is a plausible unmeasured confounder generally, but this dataset has no dietary covariate data, so we attribute no taxon’s pattern to a specific mechanism. *Akkermansia* illustrates why the coefficient-based, stability-gated definition matters: its pooled mean SHAP appears to reverse sign between cohorts (Section 3.6), but its fitted coefficient is positive and stable in all four cohorts (Supplementary Table S6)—the model never learned an inconsistent direction; the apparent reversal is a background-relative SHAP artifact, not the fitted model.

The eight taxa meeting this screen may reflect biological population heterogeneity, technical study effects, or both; the design cannot disentangle these sources, nor does the screen establish that any taxon’s pattern exceeds what a non-independent, overlapping-fold estimate could show by chance. If some directional disagreements arise from biological population differences rather than technical artifacts, technical batch correction would not necessarily resolve them; the present design cannot determine which source dominates, consistent with Section 4.2’s finding that label-blind correction left cross-cohort AUC essentially unchanged.

### 4.5 Implications for clinical translation

These results suggest that gut microbiome-based AD classifiers are not ready for clinical deployment across heterogeneous external cohorts without prospective validation. A classifier trained on Zhuang 2018 and applied to Kazakhstan achieved AUC = 0.566 for logistic regression and AUC = 0.493 for LightGBM—near chance and inadequate for diagnostic deployment.

We propose three prerequisites for meaningful clinical translation: (1) Multi-cohort harmonized training data—classifiers should train on samples from multiple geographic and demographic populations simultaneously, with cohort or population as a stratification variable, rather than training within a single cohort and expecting transfer. (2) Prospective cross-cohort validation as a publication standard—any future study reporting a gut microbiome AD classifier should validate performance on at least one independent cohort from a different geographic population before claiming clinical relevance; within-cohort AUC alone, however high, is an insufficient evidential standard. (3) Covariate-adjusted models—diet, geographic origin, antibiotic history, and other known microbiome-modulating covariates should be measured and accounted for through study design, stratification, covariate adjustment, or sensitivity analysis, reducing the influence of population-specific compositional baselines.

Multi-cohort harmonized collection has succeeded in other biomarker domains (e.g., UK Biobank for genomic predictors, ADNI for neuroimaging). A parallel initiative—a consortium of AD microbiome studies committed to harmonized collection protocols and prospective cross-cohort sharing—would be the most direct route to a clinically actionable microbiome diagnostic.

### 4.6 Limitations

Several limitations of this study should be acknowledged explicitly.

#### 4.6.1 Cohort search and selection

The search strategy (Section 2.1; Supplementary Note S1) identified five publicly available cohorts but not exhaustive of all existing AD microbiome studies, and additional non-Asian cohorts would strengthen the conclusions. Diagnostic ascertainment is not equivalent across the source studies. The four labeled cohorts used a clinical AD diagnosis, according to each study’s own protocol (Section 2.1), while Kim/KBASE 2022 (PERMANOVA-only) used amyloid-PET positivity in cognitively normal preclinical participants—a fundamentally different case definition (Supplementary Note S1)—and even the four labeled cohorts do not share identical diagnostic instruments or thresholds. This heterogeneity can itself contribute to cross-cohort transportability differences independently of microbiome biology and is not fully separable from technical batch effects here; we do not assume the four cohorts are diagnostically interchangeable.

#### 4.6.2 Mixed single-end and paired-end sequencing

Kazakhstan used single-end NovaSeq reads while all other cohorts used paired-end MiSeq 2 × 300 bp, yielding lower taxonomic resolution and different fitted error models—a technical asymmetry not disentangled from biological population differences here.

#### 4.6.3 China overrepresentation

Three of the four labeled cohorts are Chinese, so the cross-cohort experiment is primarily a China-internal transfer (Zhuang ↔ Ling ↔ Li 2019) plus transfer to/from a single Central Asian cohort. A more geographically balanced design would be needed to generalize the conclusions further.

#### 4.6.4 Partial SRA deposit for Li 2019

*Li 2019’s near-perfect within-cohort AUC cannot be attributed to biology over a technical confound*. A sequencing-metadata field is exactly collinear with diagnosis in this cohort, statistically unresolvable from the available data (Section 4.3; Supplementary Table S14; Supplementary Note S4); this does not extend to the LOCO or pairwise-transfer results, which involve classifiers trained on other cohorts.

*Modest per-cohort sample sizes limit precision*. Cohorts range from *n* = 60 (Li 2019) to *n* = 171 (Ling 2020), reflected in wide 95% CIs throughout (Tables 1, 2; Supplementary Table S1) and in four of eight within-cohort-vs-LOCO differences not reaching formal significance despite a consistent direction. We report confidence intervals rather than a retrospective observed-power calculation, which would add no information beyond them; a non-significant comparison should be read as imprecise, not as evidence of no true difference.

*Common (fixed) genus universe retained for secondary descriptive/harmonization analyses*. Batch correction, SHAP/coefficient stability, and PERMANOVA use the fixed 396-genus schema rather than the primary training-only pipeline (Section 2.5.1), because those analyses require one common feature space across cohorts; they should be interpreted only for their own descriptive/harmonization question, not as generalization estimates. The two pipelines agree closely for within-cohort/LOCO AUC (Supplementary Table S4) but disagree materially for one pairwise-transfer cell (Supplementary Table S1), disclosed rather than smoothed over (Section 3.3).

*PERMANOVA cannot decompose cohort into technical and biological sources, and the cohort-versus-diagnosis disparity is sensitive to which cohorts are compared*. “Cohort” here is a composite variable bundling sequencing platform, library preparation, recruitment site, diagnostic ascertainment, and unmeasured dietary/lifestyle differences that PERMANOVA and betadisper cannot separate (Section 2.6). As shown in Section 3.4, the ~12-fold disparity across all four labeled cohorts fell to ~2.6-fold when restricted to the three more technically comparable Chinese paired-end MiSeq cohorts, so this ratio depends substantially on which cohorts are compared; readers should not extrapolate the ~12-fold figure elsewhere.

*Multiple comparisons are handled differently across analyses, by design*. We distinguish and do not apply one blanket correction across three categories: (1) explicit multiplicity-corrected tests—the 10 pairwise beta-dispersion comparisons (Holm/BH; Section 2.6; Supplementary Table S12); (2) empirical-null significance tests, each a single pre-specified comparison against a purpose-built permutation null (Jaccard overlap, Section 2.8; Supplementary Tables S7, S13; Li 2019 label permutation, Section 2.4.1; Supplementary Table S9), not a multiple-comparisons family; and (3) descriptive, non-inferential screens—the coefficient-sign-stability screen (Section 2.8, Section 4.4), which receives no *p*-value or multiplicity correction because its 10 outer-CV folds are not independent replications. A blanket correction across all three would be inapplicable to category 3 and would not change any category-2 conclusion; we instead state which category each result belongs to.

*Jaccard overlap significance depends on the null model chosen, and one finding reverses*. LightGBM’s cross-cohort overlap of top-predictors was significant under a shared-genus-pool null but not under a per-cohort prevalence-restricted null (Section 3.6); we report both rather than just the more favorable one. Logistic regression’s overlap was significant under both nulls and is the more robust finding.

*Batch correction: two designs, both transductive, neither prospective*. Both the label-blind (primary) and label-informed (exploratory) designs (Section 2.7) estimate batch-effect parameters using the eventual held-out cohort’s own feature values, and neither should be extrapolated to prospective deployment. The label-informed design’s results (including the severe Kazakhstan collapse under label-informed ComBat-seq, Section 3.5) should not be interpreted as evidence about batch correction’s effect on biological signal in general, being substantially confounded by the design itself (Section 4.2).

*Repeated cross-validation is a fold-stability check, not independent replication* (Section 2.4.1; see the technical-confounding bullet above for what it cannot establish). Separately, the formal paired bootstrap (Section 2.5.2, Section 3.3) found that four of eight cohort–model pairs (Zhuang 2018 and Kazakhstan, both models) did not reach a 95% CI excluding zero despite all eight showing a drop in the same direction—one more non-significant pair than under the common-universe pipeline (Supplementary Table S10).

*Cross-sectional design*: Causal inference about microbiome–AD relationships is not supported; all language refers to associations and predictive performance.

*SHAP and coefficient-direction interpretability*: Logistic regression direction is defined by the fitted coefficient sign, not mean signed SHAP, and is conditional on L2 regularization (below) and CLR-feature collinearity. The eight genera meeting the stability screen are empirical patterns for follow-up, not confirmed mechanistic findings (Sections 2.8, 4.4). LightGBM has no coefficient-like direction and receives no screen; LOCO SHAP values remain importance rankings only, with no assigned direction.

*Logistic regression models use L2 (ridge), not elastic-net, regularization*: solver = “saga” uses penalty = “l2”; the configured l1_ratio has no effect under L2. Essentially, no fitted coefficient is exactly zero in the coefficient-stability analysis (Section 2.8), since L2 does not perform the sparse selection that elastic-net or L1 would.

## 5 Conclusion

We conducted a systematic cross-cohort generalization evaluation of gut microbiome-based Alzheimer’s disease classifiers spanning four labeled cohorts, with a fifth (Kim/KBASE 2022) contributing only to unsupervised compositional analyses. Using strict training-only genus selection as the primary classifier-performance pipeline (Section 2.5.1), within-cohort classifiers achieved AUC-ROC values of 0.611–0.996; the near-perfect Li 2019 result withstood fold-stability and label-permutation checks, though a technical-metadata confound cannot be excluded (Section 4.3). LOCO AUC fell to 0.506–0.812 (mean drop 0.170 for logistic regression, 0.133 for LightGBM), formally supported for four of eight cohort–model pairs (Section 3.3), and was closely consistent with a fixed common-universe reference retained for secondary analyses, with one pairwise-transfer exception disclosed rather than smoothed over (Kazakhstan→Ling 2020, LightGBM: 0.58 → 0.44). Batch correction under a label-blind design left mean LOCO AUC essentially unchanged (within 0.007 AUC) without recovering transferability; an exploratory label-informed design, which cannot estimate prospective performance, produced large, cohort-specific degradation attributable substantially to the design itself. PERMANOVA showed that cohort identity explained substantially more compositional variance than disease status (marginal R^2^: 0.172 vs. 0.014, ~12-fold), falling to ~2.6-fold when restricted to three more technically comparable cohorts—not decomposable into technical and biological sources, and not by itself explaining the batch-correction results. A cohort-identity classifier achieved macro-AUC = 0.9917, and generalization failure persisted across all cohort-exclusion sensitivity configurations. SHAP analysis found low cross-cohort taxon overlap (mean Jaccard 0.135 for logistic regression, 0.058 for LightGBM), significant for logistic regression under both null models but for LightGBM only under the less conservative one—a reversal reported as such. A pre-specified coefficient-sign-stability screen identified eight logistic-regression genera with opposite, stable direction across cohorts; no comparable screen applies to LightGBM. These findings demonstrate substantial cross-cohort generalization limitations among the evaluated 16S datasets and indicate that the two batch-correction methods tested under the label-blind transductive design did not recover transferability. Multi-cohort harmonized and prospective cross-cohort validation studies are needed before microbiome-based AD biomarkers can achieve clinical utility.

## Data Availability

Publicly available datasets were analyzed in this study. This data can be found at: Raw sequencing data are available through NCBI SRA and ENA under accession numbers PRJNA554111 (Zhuang 2018), PRJNA633959 (Ling 2020), PRJNA489760 (Li 2019), PRJNA811324 (Kaiyrlykyzy 2022), and PRJEB50447 (Kim 2022). Analysis code and processed data tables are available at: https://github.com/carhanc/microbiome-ad-generalization.
