# Supplementary material for: Cross-cohort generalization limitations in gut microbiome-based Alzheimer’s disease classifiers: evidence from four supervised cohorts with batch-correction analysis
Source: Front Aging Neurosci. 2026 Oct 22;18:1918251. doi: 10.3389/fnagi.2026.1918251 (PMC13736582; doi:10.3389/fnagi.2026.1918251)
Supplement: Supplementary file 1 [file Supplementary_file_1.pdf]

Supplementary Materials

Supplementary Tables

Table S1. Full Pairwise Cross-Cohort AUC Matrix

Pairwise AUC-ROC for single-cohort train → single-cohort test transfer experiments, under strict training-only genus selection (**primary analysis**, Section 2.5.1; Figure 2B–C). All 4×4 combinations excluding diagonal (within-cohort). Values are AUC-ROC with 95% bootstrap CIs (1,000 resamples) on the test-cohort predictions.

Table S1A — Logistic Regression (training-only, primary)

Train Test	Zhuang 2018	Ling 2020	Li 2019	Kazakhstan
Zhuang 2018	—	0.598 [0.51–0.69]	0.697 [0.55–0.84]	0.583 [0.45–0.71]
Ling 2020	0.519 [0.41–0.64]	—	0.858 [0.75–0.95]	0.601 [0.47–0.73]
Li 2019	0.594 [0.47–0.71]	0.723 [0.65–0.80]	—	0.566 [0.44–0.70]
Kazakhstan	0.545 [0.43–0.67]	0.739 [0.66–0.81]	0.650 [0.50–0.79]	—

Table S1B — LightGBM (training-only, primary)

Train Test	Zhuang 2018	Ling 2020	Li 2019	Kazakhstan
Zhuang 2018	—	0.563 [0.47–0.65]	0.642 [0.50–0.78]	0.586 [0.46–0.71]
Ling 2020	0.639 [0.53–0.75]	—	0.817 [0.70–0.91]	0.558 [0.42–0.68]
Li 2019	0.533 [0.41–0.66]	0.658 [0.57–0.73]	—	**0.432 [0.31–0.56]**
Kazakhstan	0.515 [0.39–0.64]	**0.436 [0.35–0.53]**	0.567 [0.42–0.72]	—

Notable observations: The highest pairwise AUC remains Ling 2020 → Li 2019 (LogReg=0.86, LightGBM=0.82), reflecting transfer between two large Chinese fecal cohorts. Kazakhstan → Ling 2020 LogReg also transfers reasonably (0.74). Most pairwise AUCs cluster in the interquartile range 0.55–0.65 (median 0.59; n=24 directed pairs × 2 models), consistent with the overall pattern of limited cross-cohort transferability documented in Section 3.3. **One cell is flagged explicitly**: Kazakhstan → Ling 2020 (LightGBM), bolded above, is 0.436 under training-only selection versus 0.580 under the fixed common-universe schema (Table S1, reference version below) — a Δ=−0.144, the largest shift anywhere in the training-only vs. common-universe comparison (Table S4), moving this one cell from weak positive transfer to below chance. We report this without smoothing; it does not change the overall heterogeneous-transferability conclusion, since the best- and worst-transferring pairs otherwise agree between pipelines.

Common-universe (fixed 396-genus) reference values, for comparison — identical pairwise-transfer procedure under the fixed common 396-genus schema used for this paper's secondary descriptive/harmonization analyses:

Table S1A-ref — Logistic Regression (common-universe reference)

Train Test	Zhuang 2018	Ling 2020	Li 2019	Kazakhstan
Zhuang 2018	—	0.583 [0.50–0.67]	0.696 [0.54–0.82]	0.566 [0.44–0.69]
Ling 2020	0.538 [0.41–0.66]	—	0.879 [0.77–0.96]	0.593 [0.47–0.72]
Li 2019	0.579 [0.46–0.70]	0.760 [0.68–0.83]	—	0.547 [0.43–0.67]
Kazakhstan	0.539 [0.42–0.67]	0.739 [0.66–0.81]	0.652 [0.50–0.78]	—

Table S1B-ref — LightGBM (common-universe reference)

Train Test	Zhuang 2018	Ling 2020	Li 2019	Kazakhstan
Zhuang 2018	—	0.598 [0.51–0.68]	0.604 [0.46–0.74]	0.493 [0.37–0.61]
Ling 2020	0.617 [0.49–0.74]	—	0.861 [0.77–0.94]	0.602 [0.47–0.72]
Li 2019	0.528 [0.41–0.65]	0.591 [0.50–0.68]	—	0.405 [0.28–0.53]
Kazakhstan	0.522 [0.40–0.64]	**0.580 [0.49–0.67]**	0.603 [0.45–0.75]	—

Source data (primary): `results/tables/pairwise_auc_training_only.csv`. *Source data (reference):* `results/tables/pairwise_auc.csv`.

Table S2. Per-Cohort DADA2 Processing Statistics

Statistics from DADA2 amplicon sequence variant (ASV) inference pipeline. Paired-end cohorts underwent quality filtering, error learning, denoising, merging, and chimera removal; Kazakhstan (single-end) omitted the merging step.

Cohort	Country	Mode	N Samples	Reads Input	Reads Non-chimeric	% Retention	16S Region	Platform
Zhuang 2018	China	Paired-end	86	3,260,213	2,551,870	78.3%	V3–V4	MiSeq 2×300
Ling 2020	China	Paired-end	171	8,556,209	4,100,210	47.9%	V3–V4	MiSeq 2×300
Li 2019	China	Paired-end	180	7,275,079	5,485,811	75.4%	V3–V4	MiSeq 2×300
Kim 2022	South Korea	Paired-end	78	13,514,374	6,117,631	45.3%	V3–V4	MiSeq 2×300
Kaiyrlykyzy 2022	Kazakhstan	Single-end	84	9,467,007	6,531,362	69.0%	V3–V4	NovaSeq
**Total**			**599**	**42,072,882**	**24,786,884**	**58.9%**		

Notes: (1) Reads input = reads passing initial quality cutoff prior to DADA2 filtering. (2) Retention rates for Ling 2020 (47.9%) and Kim 2022 (45.3%) are lower than typical for MiSeq 2×300 V3–V4 data; inspection of quality profiles showed elevated error rates in these cohorts' reverse reads at positions >150–180 bp, which may reflect sequencing run-specific quality variation. (3) Kazakhstan single-end processing used truncLen=250 after quality profile inspection; see Supplementary Note S2. (4) ASV count per cohort and per-sample read depth statistics not shown here; final post-harmonization genus matrix contains 396 genera across 509 fecal samples (90 blood microbiome samples from Li 2019 excluded prior to analysis).

Table S3. Full PERMANOVA Results

All PERMANOVA models used 9,999 permutations with Type III (marginal) sums of squares (vegan adonis2, `by="margin"`). Aitchison distance = Euclidean distance on CLR-transformed values. Bray-Curtis computed on relative abundances. With 9,999 permutations, the minimum attainable permutation p-value is 1/(9,999+1) = 0.0001; p-values reported below as 0.0001 indicate no permuted statistic met or exceeded the observed value (i.e., the true p-value is at or below this resolution limit, not literally less than it).

Model 1: Cohort only (all 5 cohorts, Aitchison distance)

Term	Df	Sum of Squares	R^2	F	p-value
Cohort	4	67,760.8	0.193	30.13	0.0001
Residual	504	283,347.1	0.807	—	—

Model 1B: Cohort only (all 5 cohorts, Bray-Curtis dissimilarity)

Term	Df	Sum of Squares	R^2	F	p-value
Cohort	4	20.80	0.136	19.80	0.0001
Residual	504	132.36	0.864	—	—

Model 2: Cohort + Diagnosis (4 labeled cohorts, Aitchison, marginal R^2)

Term	Df	Sum of Squares	R^2 (marginal)	F	p-value
Cohort	3	45,476.8	0.172	28.03	0.0001
Diagnosis	1	3,689.7	0.014	6.82	0.0001

Note: Marginal R^2 values for cohort and diagnosis do not sum to total R^2 because marginal effects are estimated independently (Type III SS), adjusting for the other variable. The sum of marginal R^2 values underestimates total explained variance. "Cohort" here is a composite study-of-origin variable (platform, library prep, recruitment site, diagnostic ascertainment, diet, and other unmeasured differences bundled together); this model does not, and cannot, decompose the cohort term into technical versus biological components (Section 2.6). See Table S11 for a sensitivity analysis showing this cohort-vs-diagnosis disparity is highly sensitive to which cohorts are included.

Model 3: Within-cohort Diagnosis effects (Aitchison distance)

Cohort	Term	Df	R^2	F	p-value
Zhuang 2018	Diagnosis	1	0.017	1.47	0.066
Ling 2020	Diagnosis	1	0.053	9.53	0.0001
Li 2019	Diagnosis	1	0.094	6.01	0.0001
Kazakhstan	Diagnosis	1	0.022	1.83	0.069

Li 2019 analysis restricted to fecal samples only (n=60 binary: 30 AD + 30 CN). Blood microbiome samples excluded prior to all analyses. Zhuang 2018 (p=0.066) and Kazakhstan (p=0.069) did not reach α=0.05 significance for within-cohort diagnosis effect.

Beta-Dispersion Test (Aitchison distance, all 5 cohorts)

Metric	Value
F-statistic	13.93
Numerator df	4
Denominator df	504
p-value (permutation, 9,999 perms)	0.0001
Interpretation	Within-cohort dispersions differ significantly; PERMANOVA R^2 reflects both centroid shift and spread heterogeneity

---

Table S4. Training-Only (Primary) vs. Common-Universe (Reference) Feature Selection

Genus retention (≥20% prevalence) re-derived independently within every split, using only that split's training-side samples (Section 2.5.1) — the **primary** pipeline reported in Table 1 and Table 2 — compared against the fixed common 396-genus schema (Section 2.2) used for this paper's secondary descriptive/harmonization analyses (batch correction, SHAP, PERMANOVA).

LOCO

Test Cohort	Model	Training-Only LOCO AUC (primary)	Common-Universe LOCO AUC (reference)	Δ	N Retained Genera (Training-Only)
Zhuang 2018	LogReg	0.5057	0.5041	−0.0016	370
Zhuang 2018	LGBM	0.5960	0.5646	−0.0314	370
Ling 2020	LogReg	0.6794	0.6775	−0.0019	370
Ling 2020	LGBM	0.6534	0.6735	+0.0201	370
Li 2019	LogReg	0.8122	0.8133	+0.0011	373
Li 2019	LGBM	0.7878	0.7578	−0.0300	373
Kazakhstan	LogReg	0.6092	0.5644	−0.0448	150
Kazakhstan	LGBM	0.5247	0.5905	+0.0658	150

Within-cohort nested CV

Cohort	Model	Training-Only AUC (primary)	Common-Universe AUC (reference)	Δ
Zhuang 2018	LogReg	0.6647	0.6333	−0.0314
Zhuang 2018	LGBM	0.6733	0.6349	−0.0384
Ling 2020	LogReg	0.8896	0.8648	−0.0248
Ling 2020	LGBM	0.8415	0.8618	+0.0203
Li 2019	LogReg	0.9956	0.9978	+0.0022
Li 2019	LGBM	0.9700	0.9789	+0.0089
Kazakhstan	LogReg	0.7368	0.7657	+0.0289
Kazakhstan	LGBM	0.6109	0.6943	+0.0834

The retained genus count for LOCO training-only selection is lower when Kazakhstan is excluded from feature selection (150 genera) than when it is included in the training pool (370–373 genera), reflecting that Kazakhstan alone passes the 20%-prevalence threshold for many genera the Chinese/Korean-pipeline

cohorts do not. No cohort–model combination changes qualitative classification (near-chance, intermediate, robust) between the two pipelines; the largest single within-cohort/LOCO disagreement is 0.0834 (Kazakhstan LightGBM, within-cohort). The one pairwise-transfer cell that changes qualitatively (Kazakhstan → Ling 2020, LightGBM) is reported separately in Table S1, since pairwise transfer is a distinct experiment from within-cohort/LOCO.

Source data: `results/tables/within_cohort_auc_training_only.csv`, `results/tables/loco_auc_training_only.csv`, `results/tables/pairwise_auc_training_only.csv`, `results/tables/training_only_feature_sensitivity_summary.csv`, `results/tables/training_only_feature_counts.csv`, `results/tables/training_only_feature_lists.csv` (full per-split retained-genus lists).

Table S5. Within-Cohort AUC on Batch-Corrected Data: Label-Blind vs. Label-Informed

Within-cohort nested-CV AUC (Section 2.4 procedure) computed on batch-corrected features, under both correction designs (Section 2.7). The label-blind comparison is free of diagnosis-label leakage during correction; the label-informed comparison is not (Section 3.5) and is shown only for completeness/continuity with the original analysis.

Cohort	Model	Uncorrected	ComBat-seq (label-blind)	MMUPHin (label-blind)	ComBat-seq (label-informed, exploratory)	MMUPHin (label-informed, exploratory)
Zhuang 2018	LogReg	0.6333	0.6604	0.6171	0.6647	0.6393
Zhuang 2018	LGBM	0.6349	0.6479	0.6247	0.6544	0.5765
Ling 2020	LogReg	0.8648	0.8701	0.8690	0.8754	0.8863
Ling 2020	LGBM	0.8618	0.8255	0.8689	0.8659	0.8844
Li 2019	LogReg	0.9978	0.9878	0.9900	0.9956	0.9933
Li 2019	LGBM	0.9789	0.9533	0.9600	0.9622	0.9650
Kazakhstan	LogReg	0.7657	0.6926	0.7663	0.7839	0.7879
Kazakhstan	LGBM	0.6943	0.6829	0.5621	0.7351	0.6858

Source data: `results/tables/within_cohort_auc_corrected_labelblind.csv`, `results/tables/within_cohort_auc_corrected.csv`, `results/tables/within_cohort_auc.csv`.

Table S6. Logistic-Regression Coefficient Sign Stability: Screened Candidates and the *Akkermansia* Negative Control

Per (cohort, genus), counts of positive/negative/zero fitted coefficients across the 10 outer OOF folds (Section 2.8). `sign_stability` uses the pre-specified ≥8/10-fold threshold. This is a descriptive screen, not a hypothesis test: the 10 outer folds share overlapping training data and are not independent replications, so no p-value is assigned to any row. Full table (1,584 cohort×genus rows) in

`results/tables/logreg_coefficient_stability.csv`; below, *Romboutsia* (the cleanest cohort-level pattern among the eight genera meeting the screen; Section 3.6) and *Akkermansia* (a negative-control illustration: appears to flip under the prior mean-signed-SHAP direction statistic but does not meet the coefficient-based screen; Section 3.6, Section 4.4).

Cohort	Taxon	n_positive/10	n_negative/10	Median Coefficient	Sign Stability
Zhuang 2018	Romboutsia	0	10	−0.0107	stable_negative
Ling 2020	Romboutsia	0	10	−0.1166	stable_negative
Li 2019	Romboutsia	0	10	−0.0190	stable_negative
Kazakhstan	Romboutsia	9	1	0.0109	stable_positive
Zhuang 2018	Akkermansia	10	0	0.0124	stable_positive
Ling 2020	Akkermansia	10	0	0.1181	stable_positive
Li 2019	Akkermansia	10	0	0.0709	stable_positive
Kazakhstan	Akkermansia	10	0	0.0682	stable_positive

Romboutsia is stable-negative in all three Chinese cohorts and stable-positive in Kazakhstan — the cleanest observed cross-cohort pattern among the eight genera meeting the screen. *Akkermansia*'s coefficient is stable and positive in all four cohorts — it does not meet the screen at all, despite its pooled mean SHAP value appearing to reverse sign (Section 3.6). See the full CSV for the corresponding rows for each of the eight genera meeting the descriptive screen (*Agathobacter*, *Bifidobacterium*, *Coprococcus*, *Dorea*, *Lactobacillus*, *NK4A214 group*, *Romboutsia*, *Ruminococcus gnavus group*) and for `logreg_directional_flips_stable.csv`, which lists, per candidate taxon, which cohorts carried a stable positive vs. stable negative coefficient.

Source data: `results/tables/logreg_coefficient_stability.csv`, `results/tables/logreg_directional_flips_stable.csv`.

Table S7. Matched-Null (Shared-Pool) Jaccard Overlap Across Top-N Thresholds

Observed statistic = mean of the six unique pairwise Jaccard similarities among the four labeled cohorts' top-N SHAP taxa sets. Null = 100,000 replicates, each drawing four independent random top-N gene sets from the shared 396-genus pool and averaging the same six pairwise Jaccards among them (Section 2.8). Empirical one-sided p = (1 + #{null ≥ observed}) / (n_perm + 1). See Table S13 for the prevalence-restricted (per-cohort eligible universe) sensitivity analysis, which reverses the LightGBM result reported here.

Model	Top-N	Observed Mean (6 pairs)	Null Mean	Null 95% CI	Empirical p
LogReg	10	0.0850	0.0134	[0.0000, 0.0361]	<0.0001
LogReg	**20 (primary)**	**0.1350**	0.0265	[0.0085, 0.0491]	**<0.0001**
LogReg	50	0.2296	0.0680	[0.0491, 0.0895]	<0.0001
LightGBM	10	0.0175	0.0135	[0.0000, 0.0361]	0.4494 (not significant)
LightGBM	**20 (primary)**	**0.0584**	0.0265	[0.0085, 0.0491]	**0.0048**

Model	Top-N	Observed Mean (6 pairs)	Null Mean	Null 95% CI	Empirical p
LightGBM	50	0.1430	0.0680	[0.0492, 0.0894]	<0.0001

LightGBM's overlap is significant at N=20 and N=50 but not at N=10 under this shared-pool null. Table S13 shows this result does not survive a prevalence-restricted null model.

Source data: `results/tables/jaccard_null_corrected.csv`.

Table S8. Li 2019 Repeated Nested Cross-Validation (Fold-Assignment Stability)

50 repetitions of the 10-fold outer / 5-fold inner nested-CV partition, each under a different pre-specified seed, strict training-only genus selection re-derived within every outer fold of every repetition (Section 2.4.1). This is a fold-assignment stability check on the same 60 participants, not an independent-sample replication.

Model	Historical Single-Partition AUC	Training-Only Single-Partition AUC	Repeated-CV Mean	SD	Median	P2.5	P97.5	Min	Max	N Repeats
LogReg	0.998	0.9956	0.9955	0.0027	0.9956	0.9900	0.9989	0.9889	0.9989	50
LGBM	0.979	0.9700	0.9760	0.0103	0.9767	0.9519	0.9917	0.9378	0.9944	50

Source data: `results/tables/zhu_repeated_nested_cv.csv` (per-repeat), `results/tables/zhu_repeated_nested_cv_summary.csv`.

Table S9. Li 2019 Label Permutation Test

Label-permutation test under the strict training-only pipeline and the fixed outer-fold partition (random_state=42; Section 2.4.1). Empirical p = (1 + #{null AUC ≥ observed}) / (n_perm + 1).

Model	Observed AUC (Training-Only)	N Permutations	Null Mean	Null SD	Null P2.5	Null P97.5	Empirical p	p Resolution Floor
LogReg	0.9956	1000	0.4731	0.1017	0.2800	0.6734	0.000999	0.000999
LGBM	0.9700	1000	0.4719	0.1094	0.2643	0.6878	0.000999	0.000999

No permutation among the 1,000 drawn reached the observed AUC for either model; the empirical p-value is therefore at the resolution floor for this permutation count (1/1,001) and should not be interpreted as more precise than that floor.

Source data: `results/tables/zhu_label_permutation.csv` (per-permutation), `results/tables/zhu_label_permutation_summary.csv`.

Table S10. Formal Within-Cohort vs. LOCO AUC Difference (Paired, Diagnosis-Stratified Bootstrap)

Paired, diagnosis-stratified, participant-level bootstrap (10,000 replicates; Section 2.5.2), applied to the strict training-only pipeline's out-of-fold predictions (primary analysis, Section 2.5.1; Table 1, Table 2). Δ (= AUC-within minus AUC-LOCO) is computed on identical resampled participants for both AUC values in each replicate. "Proportion Δ≤0" is the fraction of bootstrap replicates with a non-positive delta.

Cohort	Model	N	AUC Within	AUC LOCO	Observed Δ	Bootstrap Mean Δ	95% CI	Proportion Δ≤0
Zhuang 2018	LogReg	86	0.6647	0.5057	0.1590	—	[−0.015, 0.325]	0.0381
Zhuang 2018	LGBM	86	0.6733	0.5960	0.0773	—	[−0.096, 0.247]	0.1908
Ling 2020	LogReg	171	0.8896	0.6794	0.2101	—	[0.122, 0.300]	0.0000
Ling 2020	LGBM	171	0.8415	0.6534	0.1882	—	[0.095, 0.284]	0.0000
Li 2019	LogReg	60	0.9956	0.8122	0.1833	—	[0.071, 0.308]	0.0001
Li 2019	LGBM	60	0.9700	0.7878	0.1822	—	[0.069, 0.308]	0.0003
Kazakhstan	LogReg	84	0.7368	0.6092	0.1276	—	[−0.029, 0.283]	0.0559
Kazakhstan	LGBM	84	0.6109	0.5247	0.0862	—	[−0.093, 0.268]	0.1786

Four of eight pairs have a 95% CI excluding zero (Ling 2020 both models, Li 2019 both models). Four (Zhuang 2018 both models, Kazakhstan both models) show the same-signed delta with a CI that includes zero and are not formally significant at this sample size.

Source data (primary, training-only): `results/tables/within_vs_loco_auc_difference_bootstrap_training_only.csv`, `results/tables/within_cohort_auc_ci_stratified_bootstrap_training_only.csv`, `results/tables/loco_auc_ci_stratified_bootstrap_training_only.csv`.

Common-universe (fixed 396-genus) reference values, for comparison — identical bootstrap procedure applied to the fixed common-universe predictions used for this paper's secondary descriptive/harmonization analyses (Section 2.5.1):

Cohort	Model	AUC Within	AUC LOCO	Observed Δ	95% CI	Significant?
Zhuang 2018	LogReg	0.6333	0.5041	0.1293	[−0.047, 0.303]	no
Zhuang 2018	LGBM	0.6349	0.5646	0.0703	[−0.100, 0.239]	no
Ling 2020	LogReg	0.8648	0.6775	0.1873	[0.097, 0.277]	yes
Ling 2020	LGBM	0.8618	0.6735	0.1883	[0.096, 0.284]	yes
Li 2019	LogReg	0.9978	0.8133	0.1844	[0.072, 0.312]	yes
Li 2019	LGBM	0.9789	0.7578	0.2211	[0.111, 0.346]	yes

Cohort	Model	AUC Within	AUC LOCO	Observed Δ	95% CI	Significant?
Kazakhstan	LogReg	0.7657	0.5644	0.2014	[0.044, 0.357]	**yes (common-universe only)**
Kazakhstan	LGBM	0.6943	0.5905	0.1038	[−0.049, 0.255]	no

The common-universe reference analysis yields five of eight intervals excluding zero, whereas the primary strict training-only analysis yields four of eight; Kazakhstan logistic regression is the one significance classification that differs between the two pipelines (Section 3.3).

Source data (common-universe reference): `results/tables/within_vs_loco_auc_difference_bootstrap.csv`, `results/tables/within_cohort_auc_ci_stratified_bootstrap.csv`, `results/tables/loco_auc_ci_stratified_bootstrap.csv`.

Table S11. PERMANOVA Sensitivity: Chinese Paired-End MiSeq Cohorts Only (Zhuang 2018 + Ling 2020 + Li 2019)

Marginal PERMANOVA (Aitchison distance, `by="margin"`, 9,999 permutations), binary AD/CN only, restricted to the three cohorts that are more technically comparable to one another (same country, same paired-end MiSeq V3–V4 platform; Section 2.6). n=317 (Zhuang 2018=86, Ling 2020=171, Li 2019=60).

Term	Df	Sum of Squares	R^2 (marginal)	F	p-value
Cohort	2	17,532.9	0.0588	10.05	0.0001
Diagnosis	1	6,821.2	0.0229	7.83	0.0001

Compare to the full four-cohort marginal model (Table S3, Model 2): cohort R^2=0.172, diagnosis R^2=0.014 (~12-fold disparity). Restricted to this more technically comparable three-cohort subset, the disparity falls to ~2.6-fold (0.0588/0.0229). Beta-dispersion on this subset: F=7.946, p=0.0004 (9,999 permutations).

Pairwise betadisper post-hoc comparisons within this three-cohort subset (permutation p, with Holm adjustment across these three tests only — this is a separate multiplicity family from the five-cohort Table S12 below, not a subset of it):

Comparison	p (permutation, unadjusted)	p (Holm, 3 tests)	Significant after Holm correction?
Zhuang 2018 vs. Ling 2020	0.0001	0.0003	Yes
Zhuang 2018 vs. Li 2019	0.026	0.052	No
Ling 2020 vs. Li 2019	0.343	0.343	No

Zhuang 2018 differs significantly in dispersion from Ling 2020 after Holm correction; its difference from Li 2019 does not survive multiplicity correction (raw p=0.026, Holm-adjusted p=0.052 — at or above the conventional 0.05 threshold, so not called significant here). Ling 2020 and Li 2019 do not differ.

Source data: `results/tables/permanova_chinese_miseq_sensitivity.csv`, `results/tables/betadisper_chinese_miseq_sensitivity.csv`, `results/tables/betadisper_chinese_miseq_pairwise.csv`.

Table S12. Pairwise Beta-Dispersion Post-Hoc Comparisons (All Five Cohorts, Aitchison Distance)

All 10 pairwise betadisper comparisons among the five cohorts (9,999 permutations per pair), with Holm and Benjamini-Hochberg (BH) multiplicity adjustment across the 10 comparisons (Section 2.6). Sorted by unadjusted (permutation) p. The "p (parametric)" column is vegan's `permutest.betadisper(..., pairwise=TRUE)$pairwise$observed` — the two-sided p-value from a pooled-variance two-sample t-test evaluated on the observed (unpermuted) distances-to-centroid, using the asymptotic t reference distribution. It is not an F-statistic (pairwise betadisper comparisons use a t-test, not an F-test — only the omnibus test across all groups, Table S12's parent analysis, uses F) and is reported here for completeness; all significance calls in this paper use the permutation-based p-value and its Holm/BH adjustment, not this parametric column.

Comparison	p (parametric)	p (permutation, unadjusted)	p (Holm)	p (BH)
Kazakhstan–Ling 2020	1.434×10^{-8}	0.0001	0.0010	0.000333
Kim/KBASE 2022–Ling 2020	4.178×10^{-9}	0.0001	0.0010	0.000333
Ling 2020–Zhuang 2018	5.442×10^{-5}	0.0001	0.0010	0.000333
Kazakhstan–Li 2019	1.313×10^{-3}	0.0009	0.0063	0.00225
Kim/KBASE 2022–Li 2019	1.670×10^{-3}	0.0018	0.0108	0.0036
Ling 2020–Li 2019	8.277×10^{-3}	0.0084	0.0420	0.0140
Kazakhstan–Zhuang 2018	1.047×10^{-2}	0.0101	0.0420	0.01443
Kim/KBASE 2022–Zhuang 2018	2.507×10^{-2}	0.0216	0.0648	0.0270
Li 2019–Zhuang 2018	2.213×10^{-1}	0.2197	0.4394	0.2441
Kazakhstan–Kim/KBASE 2022	3.487×10^{-1}	0.3544	0.4394	0.3544

Seven of ten pairwise comparisons reach Holm-adjusted significance ($p<0.05$); the largest significant Holm-adjusted p-value is 0.0420 (Ling 2020–Li 2019 and Kazakhstan–Zhuang 2018). Ling 2020 differs significantly in dispersion from every other cohort (all Holm-adjusted $p \le 0.0420$) and is the cohort driving most of the overall significant dispersion difference (Section 3.4). Li 2019 vs. Zhuang 2018, Kim/KBASE 2022 vs. Zhuang 2018, and Kazakhstan vs. Kim/KBASE 2022 are not significantly different in dispersion after Holm adjustment (all Holm $p \ge 0.065$).

Source data: `results/tables/betadisper_pairwise.csv`.

Table S13. Prevalence-Restricted Jaccard Null Sensitivity

Per-cohort eligible universe = genera reaching ≥20% prevalence within that cohort's own AD/CN-labeled samples (eligible universe sizes: Zhuang 2018=131, Ling 2020=126, Li 2019=115, Kazakhstan=356). 100,000 replicates per (model, N) combination (Section 2.8). This supersedes the shared-pool null (Table S7) as the more realistic sensitivity check; the two null models disagree for LightGBM.

Model	Top-N	Observed Mean (6 pairs)	Null Mean	Null 95% CI	Empirical p
LogReg	10	0.0850	0.0260	[0.0000, 0.0624]	0.001670
LogReg	**20 (primary)**	**0.1350**	0.0522	[0.0261, 0.0841]	**0.000030**
LogReg	50	0.2308	0.1422	[0.1140, 0.1737]	0.000010
LightGBM	10	0.0175	0.0260	[0.0000, 0.0624]	0.799592 (not significant)
LightGBM	**20 (primary)**	**0.0629**	0.0522	[0.0261, 0.0841]	0.240838 (not significant)
LightGBM	50	0.1665	0.1422	[0.1140, 0.1737]	0.061309 (not significant)

Logistic regression remains significant at all three thresholds under this more realistic null. LightGBM is not significant at any of N=10/20/50 under this null, reversing the shared-pool-null result at N=20/50 (Table S7). We report this reversal directly: LightGBM's apparent cross-cohort top-predictor overlap is not distinguishable from chance once the null model accounts for each cohort's own genus-prevalence structure.

Source data: `results/tables/jaccard_prevalence_restricted_null.csv`.

Table S14. Li 2019 Technical Metadata Assessment (n=60 AD/CN Supervised Subset)

Every non-identifier SRA RunInfo field for the Li 2019 fecal AD/CN subset (Section 4.3; Note S4), tabulated against diagnosis. To assess whether sequencing-batch/diagnosis confounding could contribute to this cohort's near-perfect within-cohort AUC, every usable technical field was checked directly against diagnosis.

Field	Observed Values (n=60)	Relationship to AD/CN	Usable as Independent Batch Variable?	Interpretation
`avgLength`	600 bp (n=30), 602 bp (n=30)	**Exact**: all 600 bp runs are AD, all 602 bp runs are CN, zero exceptions	No — exactly collinear with diagnosis	Cannot be used as an adjustment covariate (Note S4); the strongest technical/diagnosis association found
`LibraryLayout`	PAIRED (constant)	Constant across all 60 samples	No — no variation	Not a candidate confound
`Platform`	ILLUMINA (constant)	Constant	No	Not a candidate confound
`Model` (instrument)	Illumina MiSeq (constant)	Constant	No	Not a candidate confound
`LibraryStrategy`	AMPLICON (constant)	Constant	No	Not a candidate confound
`LibrarySelection`	PCR (constant)	Constant	No	Not a candidate confound
`CenterName`	Ruijin Hospital Affiliated to Shanghai Jiao Tong University	Constant	No	Not a candidate confound

Field	Observed Values (n=60)	Relationship to AD/CN	Usable as Independent Batch Variable?	Interpretation
	School of Medicine (constant)			
`ReleaseDate`	6 distinct timestamps, all within the same calendar day/hour	Strong but not exact (e.g., one timestamp bucket = 15 AD / 3 CN)	Partially — concordant with the `avgLength` grouping but not itself exactly collinear	Consistent with, not independent proof of, a submission-batch structure; not over-interpreted further
Run accession (`Run`, SRR#) ordering	Ascending SRR7811020–SRR7811186	Largely block-clustered by diagnosis, with exceptions (e.g., a few CN runs interspersed within an otherwise AD-dominated block)	No — imperfect, and accession order is not a validated technical variable	Consistent with samples having been submitted in largely, not perfectly, separate diagnosis-grouped batches
`spots`, `bases`, `size_MB`	Continuous, sample-specific	Overlapping distributions; AD systematically lower mean than CN	No — overlapping, not a clean grouping variable	Corroborates the `avgLength`-linked grouping rather than constituting an independent confound
Lane / plate / extraction-batch / sequencing-run identifier	**Not present in the deposited metadata**	N/A	N/A	No field of this kind is included in this cohort's public SRA export; a physical batch, lane, or extraction group cannot be identified from the data available to us

Source data: `data/raw/shanghai2022/metadata.tsv` (SRA RunInfo export); crosstabulation restricted to the 60 fecal AD/CN runs used in supervised analyses.

Supplementary Figures

Figure S1. Li 2019: Fecal-Only Within-Cohort AUC

Caption: Within-cohort AUC-ROC for Li 2019 fecal samples only (n=60 binary: 30 AD + 30 CN), estimated using standard StratifiedKFold 10-fold nested cross-validation under the fixed common 396-genus universe (common-universe reference value; the primary training-only value in Table 1 is 0.996/0.970). Blood microbiome (B_*) samples were excluded from all analyses; each participant appears exactly once in the fecal-only dataset, making standard StratifiedKFold appropriate. Logistic regression achieves AUC=0.998 [0.99–1.00] and LightGBM achieves AUC=0.979 [0.94–1.00] under this common-universe schema. The near-perfect within-cohort AUC should be interpreted cautiously given the small sample size (30 per class) and the technical-metadata assessment in Section 4.3; see Section 3.2 for discussion. Error bars = 95% CI from the diagnosis-stratified, participant-level bootstrap (10,000 replicates; Section 2.5.2).

Source data: results/model_outputs/within_cohort_cv/shanghai2022_*.csv

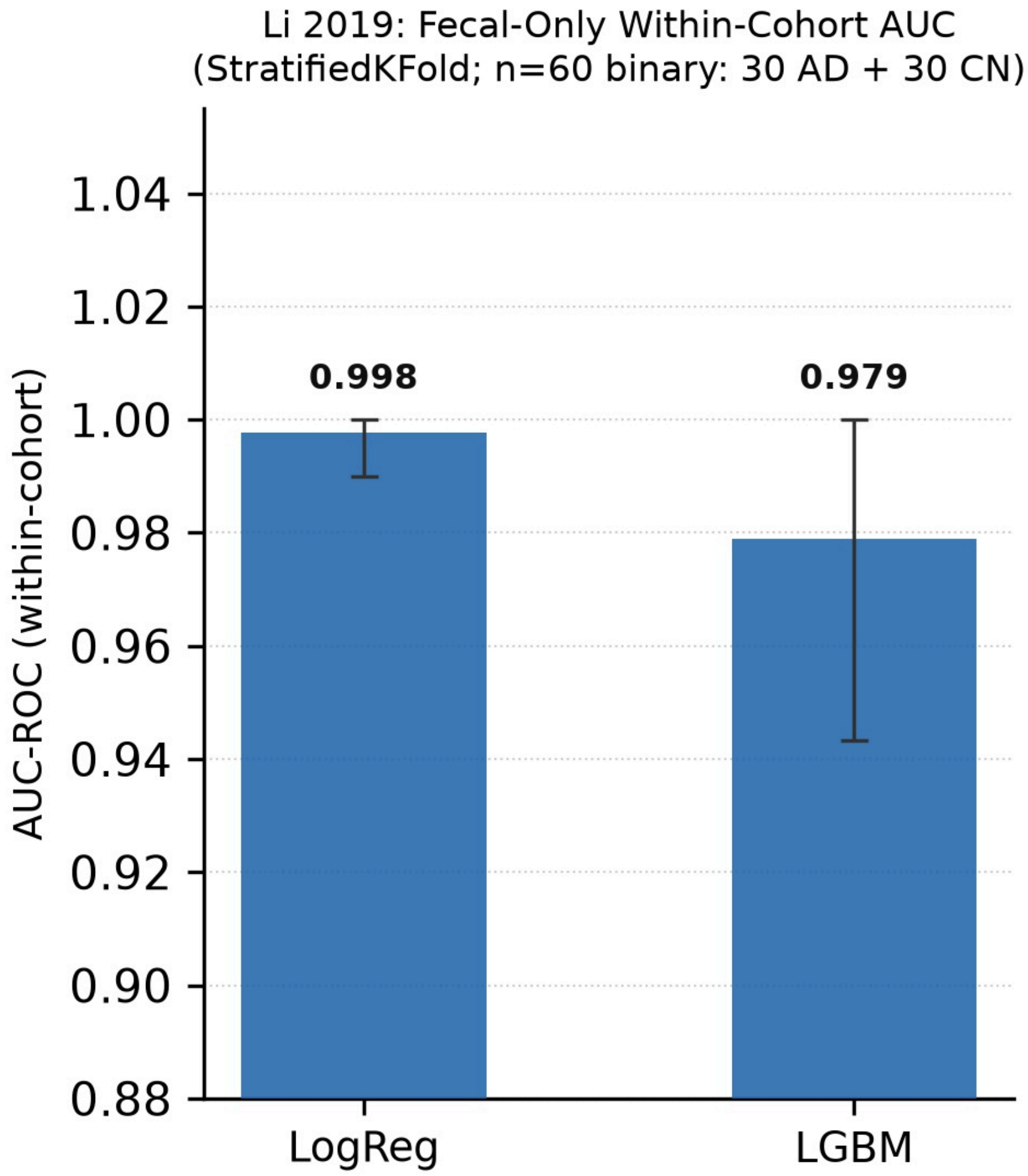

Source figure: `results/figures/final_supplementary/Figure_S1.jpg`

Figure S2. Top-15 SHAP Taxa Per Cohort (LightGBM, Out-of-Fold)

Caption: Horizontal bar charts showing mean |SHAP| value (CLR units) for the top-15 genera by importance in each of the four labeled cohorts under out-of-fold LightGBM evaluation. Bars are a single neutral color and show feature-importance magnitude only — **no AD/CN direction is assigned**, because a tree ensemble has no single coefficient-like global direction and LightGBM's SHAP-feature relationships may be nonlinear or non-monotonic (Section 2.8). Top taxon per cohort (by |SHAP| magnitude only): Lachnoclostridium (Zhuang 2018, |SHAP|=0.560), Akkermansia (Ling 2020, |SHAP|=0.947), Bacteroides (Li 2019, |SHAP|=1.243), Castellaniella (Kazakhstan, |SHAP|=0.614). Mean pairwise Jaccard similarity at top-20: 0.058, significant against the shared-pool null (p=0.0048 at N=20; not significant at N=10, p=0.45; Table S7) but not significant at any N against the prevalence-restricted null (Table S13). **No LightGBM directional-flip claim is made anywhere in this paper**, because a tree ensemble has no single coefficient-like global direction (Section 2.8).

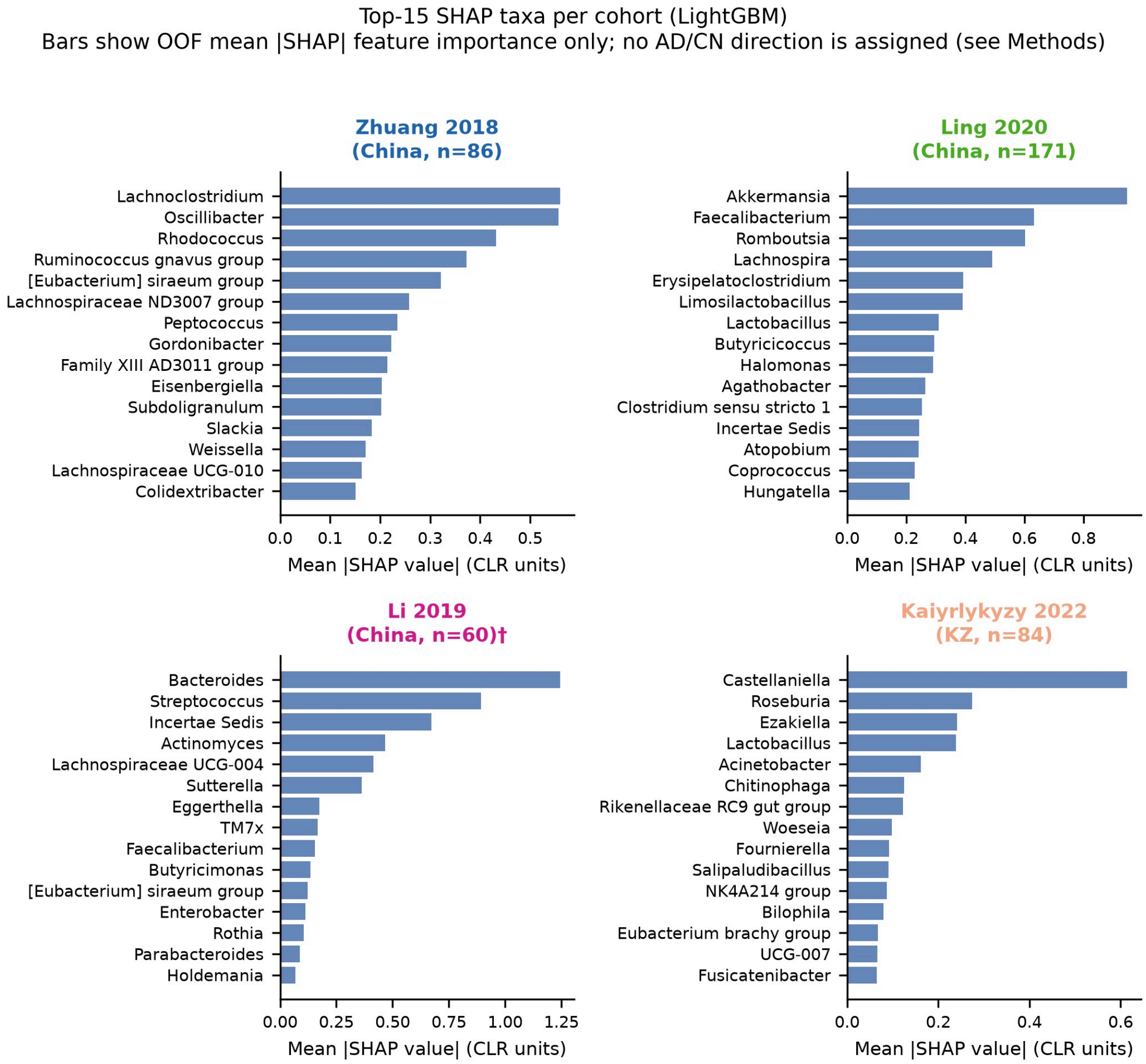

Figure S2

Source figure: `results/figures/final_supplementary/Figure_S2.jpg`

Figure S3. Sensitivity Analysis: LOCO AUC Under Cohort Exclusion

Caption: Sensitivity analysis: LOCO AUC under three cohort exclusion configurations. Each panel shows the 3-cohort LOCO experiment with one cohort excluded from the full analysis. Left: excluding Kazakhstan (Zhuang+Ling+Li trained, each held out in turn). Center: excluding Zhuang 2018. Right: excluding Ling 2020. Error bars = 95% bootstrap CI (1,000 resamples on the test-set predictions; the non-stratified procedure described in Section 2.5, not the diagnosis-stratified 10,000-replicate procedure used for the main four-cohort LOCO results). Generalization degradation persisted across all three prespecified cohort-exclusion configurations.

Source data: `results/tables/sensitivity_loco.csv`

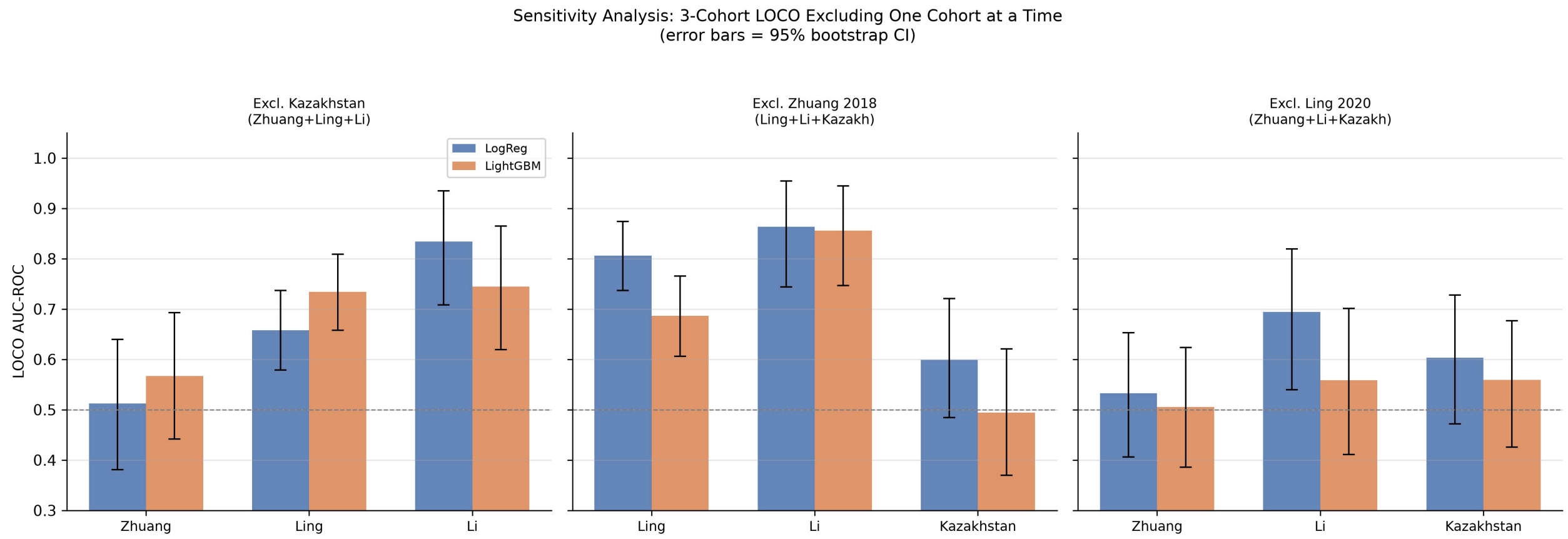

Source figure: `results/figures/final_supplementary/Figure_S3.jpg`

Figure S4. Batch Correction, Label-Informed Design (Exploratory Sensitivity)

Caption: LOCO AUC-ROC under the label-informed transductive batch-correction design (ComBat-seq/MMUPHin fit using every sample's own true diagnosis label; Section 2.7), shown for (**A**) logistic regression and (**B**) LightGBM. Error bars = 95% bootstrap CI (1,000 resamples on the test-set predictions, the same non-stratified procedure used for Table 3/Figure 3; not part of the diagnosis-stratified 10,000-replicate regeneration in Section 2.5.2). This design is retained as an **explicitly-labeled exploratory sensitivity analysis only**, because a correction step that is fit using every sample's own true diagnosis label cannot estimate prospective external-validation performance, and because the resulting within-cohort AUC on corrected data cannot be used as evidence that genuine, correction-independent biological signal survived (Section 3.5). The main-text Figure 3 shows the label-blind design (no diagnosis information used during correction), which is the paper's primary batch-correction result. Note the severe, cohort-specific degradation visible here (e.g., Kazakhstan logistic regression LOCO AUC=0.308) that is **not** reproduced under the label-blind design (Kazakhstan label-blind LOCO AUC=0.560, closely matching the uncorrected value of 0.564) — indicating this degradation was driven substantially by the label-informed design itself.

Source data: `results/tables/auc_comparison_table.csv`, `results/tables/loco_auc_corrected.csv`

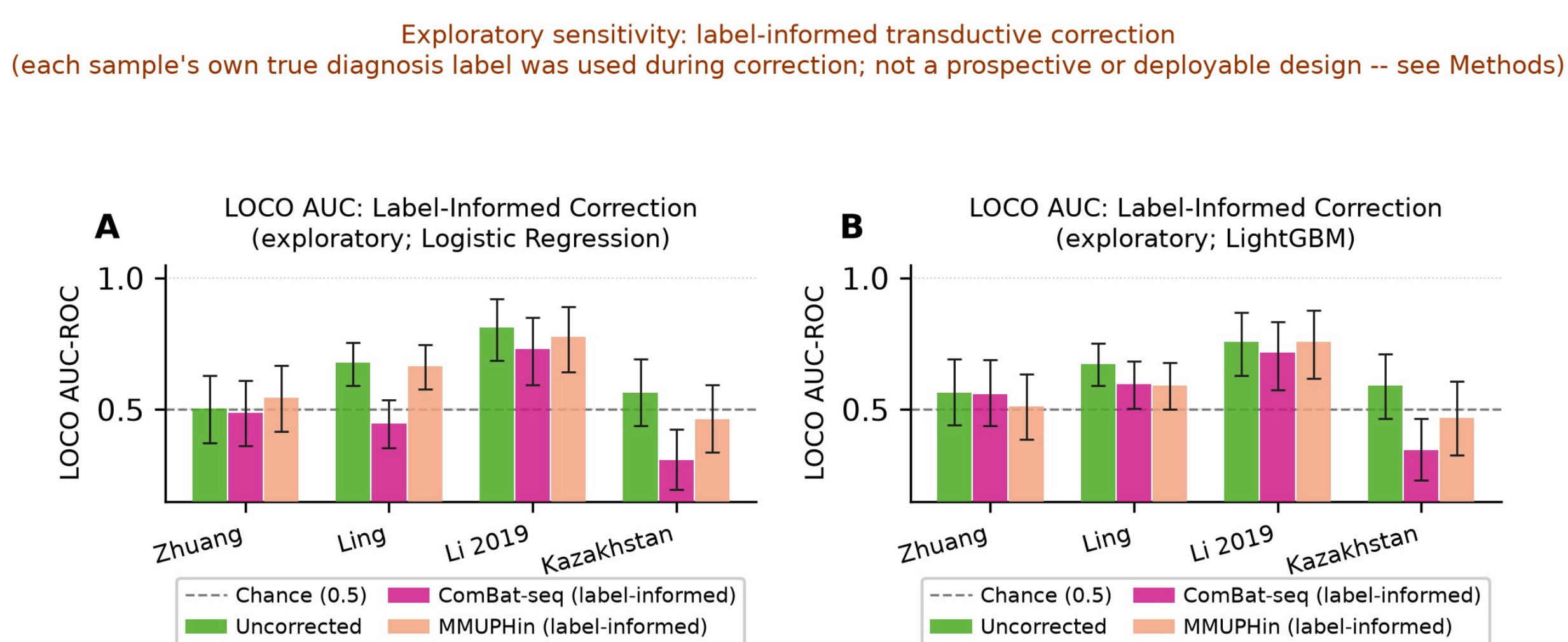

Source figure: `results/figures/final_supplementary/Figure_S4.jpg`

Figure S5. Li 2019 Robustness: Repeated Cross-Validation and Label Permutation

Caption: Robustness analyses for Li 2019's near-perfect within-cohort AUC (Section 2.4.1, Section 3.2), both run under the strict training-only feature-selection pipeline. Top row: distribution of pooled-OOF AUC across 50 repetitions of the nested-CV partition (different pre-specified seed per repetition) for logistic regression (left) and LightGBM (right), with the single-partition training-only AUC marked. Bottom row: null distribution of pooled-OOF AUC under label permutation (labels shuffled, class balance preserved; all label-dependent model fitting and hyperparameter tuning re-run inside every outer fold for each permutation, while fold-specific genus selection and CLR construction — which depend only on fold membership, not labels — were precomputed once per partition and reused across permutations), with the observed single-partition AUC marked and the empirical p-value shown in the panel title. The repeated-CV panels quantify sensitivity to fold assignment on the same 60 participants (not independent replication); the permutation panels are the formal test of whether the observed AUC exceeds a chance-label null.

Source data: `results/tables/zhu_repeated_nested_cv.csv`, `results/tables/zhu_label_permutation.csv`.

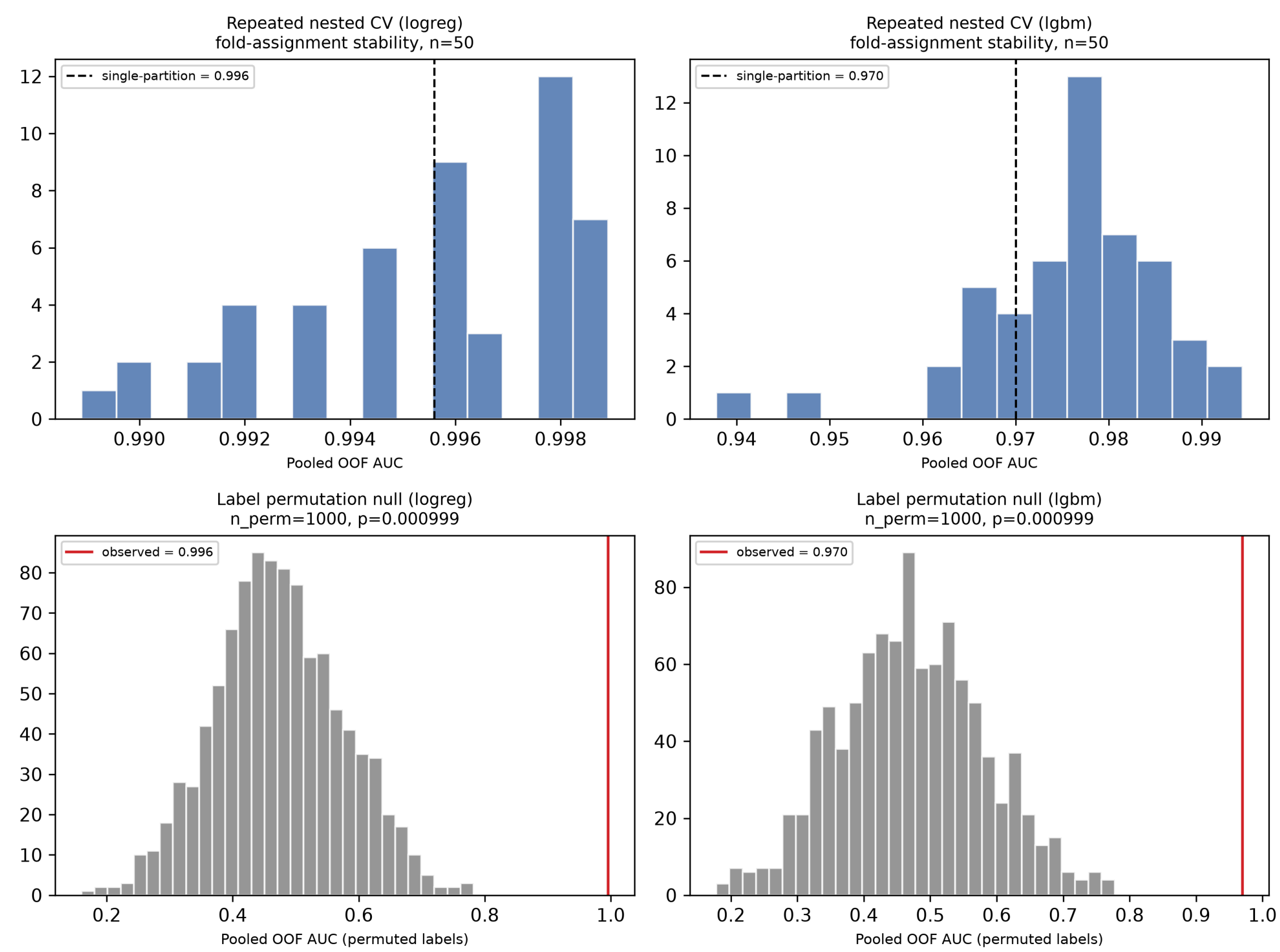

Source figure: `results/figures/final_supplementary/Figure_S5.png`

Supplementary Notes

Note S1. Kim/KBASE 2022 (PRJEB50447) — IRB Restriction on Per-Sample Diagnosis Labels

Summary: The Kim/KBASE 2022 cohort (78 participants; South Korea; 18 amyloid-PET positive preclinical AD, 60 cognitively normal) was excluded from all supervised classifier analyses because per-sample amyloid-PET status and group labels are not publicly deposited in any data archive.

Documentation of exhaustive metadata search conducted 2026-06-20:

The following metadata sources were queried for per-sample diagnosis labels:

1. **ENA Portal filereport** (https://www.ebi.ac.uk/ena/portal/api/filereport?accession=PRJEB50447&result=read_run&fields=all): All sample fields including `sample_alias`, `sample_title`, `scientific_name`, `sample_description` — no diagnostic labels present. Fields contain sequencing run identifiers only.
2. **ENA BioSample XML**: Retrieved individual BioSample records via ENA Browser. Attributes present: collection date, geographic location, library strategy/layout — no diagnosis, amyloid status, or clinical phenotype fields.
3. **NCBI SRA runinfo** (via `esearch -db sra -query "PRJEB50447" | efetch -format runinfo`): Confirmed SRA runinfo does not contain phenotype data beyond SRA-standard fields. Disease column is empty for all runs.
4. **Sample alias and title fields**: `sample_alias` values are coded identifiers (e.g., "KBASE-CN-001") without diagnostic information inferrable from the coding scheme.

Published paper finding: The corresponding publication (Kim et al., 2022; PRJEB50447) reports group-level counts (18 amyloid-positive preclinical AD; 60 cognitively normal) and notes that per-sample data are withheld under institutional IRB protocol. The amyloid-PET classifications are considered clinical data and are protected. The published paper does not provide a supplementary file with per-sample labels.

Consequence for analysis: Kim/KBASE 2022 was included only in PERMANOVA variance decomposition (Phase 4), where cohort identity — not per-sample diagnosis — is the grouping variable of interest. All supervised classifier training, evaluation, batch correction, and SHAP analyses were restricted to the four labeled cohorts (Zhuang 2018, Ling 2020, Li 2019, Kazakhstan).

Additional caveat: Even if per-sample labels were available, direct comparison with the other four cohorts would require caution. Kim/KBASE 2022 uses amyloid-PET positivity as the case definition (preclinical AD — cognitively normal by neuropsychological testing but amyloid-positive), while the other four cohorts use clinical AD diagnosis (cognitive impairment criterion). These phenotype definitions are not equivalent.

Recommendation for future analyses: Researchers wishing to include Kim/KBASE 2022 in supervised analyses should contact the study team (KBASE Consortium / corresponding author of Kim et al., 2022) to request a data use agreement for per-sample amyloid-PET status.

Cohort search strategy and full inclusion/exclusion record: The cohort identification search (Section 2.1) queried NCBI SRA and EBI-ENA for studies deposited before January 2025, using the following search string in the SRA search interface: `("Alzheimer's disease" OR "Alzheimer disease" OR "mild cognitive impairment" OR "MCI" OR "dementia") AND ("gut microbiome" OR "gut microbiota" OR "intestinal microbiome" OR`

`"16S rRNA") AND ("Homo sapiens")`. For supervised analyses, inclusion required: (1) per-sample diagnosis labels (AD, MCI, or CN) accessible in public metadata; (2) raw FASTQ files or quality-filtered reads deposited in a public archive; (3) a minimum of 40 participants per cohort. Studies were excluded from supervised analyses if per-sample labels were IRB-restricted, if data had not been publicly released at the time of analysis, or if samples represented a tissue compartment other than fecal microbiome as the primary analyte; shotgun metagenomic cohorts meeting all other criteria were identified but excluded to avoid mixing amplicon and whole-genome sequencing platforms within the same analysis. Beyond the Kim/KBASE 2022 IRB restriction documented above, one further identified cohort was excluded on public-availability grounds: **ALBION 2025** (Maraki et al., *Journal of Alzheimer's Disease*, 2025; PRJNA1297934), a Greek cohort of 99 participants (50 MCI, 49 CN; V3–V4) — the only European 16S cohort identified in the search — was confirmed as the correct accession from the published paper, but NCBI BioProject reported the deposit as not yet publicly released as of the search date. ALBION was therefore excluded from the active cohort set; it remains the most direct candidate for a future sixth cohort should the data be released.

Note S2. Kazakhstan Single-End Processing Rationale

Summary: The Kazakhstan cohort (PRJNA811324; Kaiyrlykyzy et al., 2022) was confirmed as single-end sequencing and processed using the DADA2 single-end workflow. This document explains the processing decisions made.

Confirmation of single-end format: After downloading all 84 samples with `fasterq-dump`, output files were `SRR*_1.fastq` only (no `SRR*_2.fastq` files). This was independently confirmed by inspecting SRA metadata (LibraryLayout=SINGLE). The original publication describes NovaSeq sequencing of V3–V4 amplicons.

Truncation length decision (250 bp): Quality profile inspection of Kazakhstan reads showed a characteristic NovaSeq quality pattern: high-quality bases through approximately position 250 bp, with rapid quality decline thereafter. MiSeq 2×300 bp paired-end reads for V3–V4 have effective merged read lengths of approximately 400–450 bp; single-end NovaSeq reads truncated at 250 bp cover a shorter portion of the amplicon. The truncLen parameter was set to 250 bp after visual inspection of perposition quality score profiles across 10 representative samples.

Implications for taxonomic resolution: Single-end reads provide lower taxonomic resolution than paired-end merged reads for the same amplicon region, because the additional bases from the reverse read improve amplicon coverage and reduce ambiguous taxonomic assignments. This means genus-level assignments for Kazakhstan may be slightly less accurate than for the four paired-end cohorts, and species-level analysis would be more affected than genus-level. We acknowledge this as a limitation of the mixed-library-design dataset.

DADA2 workflow difference: The single-end workflow did not include `mergePairs()`. The full single-end DADA2 pipeline applied: `filterAndTrim()` → `learnErrors()` → `dada()` → `makeSequenceTable()` → `removeBimeraDenovo()`. Error learning was performed independently on Kazakhstan samples; sharing error models across cohorts would be inappropriate because error profiles differ by sequencer and run.

Interaction with batch correction: The single-end/paired-end asymmetry is an additional source of between-cohort technical heterogeneity that may not be fully removed by generic post-hoc batch adjustment, because sequencing mode, read length, and sequencing chemistry can broadly affect abundance estimates. This asymmetry remains a limitation of analyses including Kazakhstan.

Note S3. LOCO SHAP Direction Not Reported

No figure or table showing a signed AD/CN direction for LOCO SHAP values is included in this paper. Mean signed SHAP is not used here as a global fitted-direction statistic, because it is background-relative and does not identify the sign of the fitted logistic coefficient (Section 2.8), and this is especially severe for LOCO SHAP specifically: the SHAP background (the three pooled training cohorts) and the evaluation set (the held-out cohort) are drawn from systematically different populations by construction, so the sign of a LOCO mean SHAP value is confounded with the raw cross-cohort compositional difference quantified by PERMANOVA (Section 3.4), independent of anything the model learned. No coefficient-based replacement direction is offered for LOCO SHAP either, because LOCO models are refit on pooled multi-cohort training data each time and a single stable-fold coefficient analysis analogous to Section 2.8's within-cohort procedure is not part of this design.

LOCO SHAP **feature importance** (mean |SHAP|, magnitude only, no direction) remains available and is unaffected by this issue: see `results/tables/shap_loco_importance.csv`. For reference, the top-importance genus per held-out cohort is: Zhuang 2018 — *Akkermansia* (logistic regression |SHAP|=0.470); Ling 2020 — *Subdoligranulum* (|SHAP|=0.269); Li 2019 — *Akkermansia* (|SHAP|=0.372); Kazakhstan — *Christensenellaceae R-7 group* (|SHAP|=0.368); no genus ranked first across all four held-out conditions for either model (Section 3.6).

Note S4. Li 2019 Technical-Metadata Confounding Assessment

Motivation. Li 2019's within-cohort AUC (logistic regression 0.996, LightGBM 0.970 under the primary training-only pipeline; Table 1) is the highest of the four labeled cohorts, estimated from only n=60 (30 AD, 30 CN). Because this cohort showed near-perfect within-cohort discrimination, we assessed whether available technical metadata showed diagnosis-associated structure that could contribute to this result rather than biology. This note documents a direct check of every usable technical field in this cohort's deposited SRA metadata against diagnosis, restricted to the 60 fecal AD/CN runs (full field-by-field results in Supplementary Table S14).

What we found. Of 47 raw SRA RunInfo fields, most are either sample-unique identifiers (accession numbers, hashes) or constant across all 60 samples (`LibraryLayout`, `Platform`, `Model`, `LibraryStrategy`, `LibrarySelection`, `CenterName`) and so cannot confound diagnosis. Among the remaining fields: `avgLength` (SRA-reported mean read length) is **exactly** associated with diagnosis — every one of the 30 AD runs is recorded at 600 bp and every one of the 30 CN runs at 602 bp, with no exceptions. `ReleaseDate` and run-accession (`Run`, SRR#) ordering show a concordant but non-exact grouping pattern (e.g., one release-timestamp bucket contains 15 AD and 3 CN runs; a handful of CN runs are interspersed within an otherwise AD-dominated accession block). `spots`, `bases`, and `size_MB` are continuous and overlapping between diagnosis groups, but AD runs show a systematically

lower mean than CN runs, consistent with the `avgLength` split. **No lane, plate, extraction-batch, or sequencing-run identifier is present anywhere in this cohort's deposited public metadata**, so we cannot identify a physical batch directly; `avgLength` is simply the strongest candidate marker available in the public export.

Mechanistic interpretation. We inspected the actual DADA2 invocation used for this cohort (`scripts/utils/dada2_pipeline.R`; Section 2.2): all paired-end reads in a cohort are truncated to one fixed length (approximately 240 bp forward / 200 bp reverse for Li 2019), applied uniformly regardless of a given sample's raw `avgLength`. This means the literal 2 bp raw-read-length difference between the two `avgLength` groups is discarded during truncation, before ASV inference — it cannot itself survive into the genus-level count table as a read-length artifact. We therefore distinguish two readings: (a) `avgLength` *directly causing* the classifier's discrimination — not mechanistically plausible given post-truncation processing; versus (b) `avgLength` *acting as a marker* for some other diagnosis-correlated technical grouping (e.g., a distinct sequencing run, reagent lot, or library-prep batch) whose other consequences (subtle error-profile or depth differences) could still survive into the processed data — this reading is not ruled out.

Why we cannot resolve this further with the available data. Diagnosis and `avgLength` are exactly collinear in this 60-sample subset (every 600 bp run is AD; every 602 bp run is CN): there is zero within-`avgLength`-group diagnostic variance to test against. No regression adjustment, stratified analysis, or covariate-control procedure can separate a diagnosis effect from an exactly collinear covariate — this is a structural identifiability limit of the deposited data, not a modeling choice we made. We did not attempt an adjusted analysis for this reason, and no other deposited field provides an independent, non-collinear technical grouping variable to attempt one against.

What this does and does not license us to claim. Supportable: a technical metadata field is exactly associated with diagnosis in this subset, other technical/submission metadata are concordantly (though not perfectly) grouped, and the public data cannot separate a technical from a biological explanation for this cohort's near-perfect classification. Not supportable: that `avgLength` causes the classification (no plausible mechanism survives truncation), that we have identified a specific physical batch (no such field is deposited), or that this proves the result is a technical artifact rather than genuine biology (we simply cannot tell). We report this as a disclosed, unresolved limitation (Section 4.3, Section 4.6), not a demonstrated confound.

Interpretation of existing robustness checks. The repeated nested-CV (50 partitions) and 1,000-permutation label test (Section 2.4.1, Section 3.2) establish that this cohort's AUC is stable to fold reassignment and far exceeds a chance-label null. Neither check bears on the question addressed by this note: a reproducible, diagnosis-correlated technical confound would be exactly as stable across fold reassignment and exactly as distinguishable from a chance-label permutation null as genuine biological signal would be. These checks and this note answer different questions and should not be conflated.

Source data: `data/raw/shanghai2022/metadata.tsv` (SRA RunInfo export); `scripts/utils/dada2_pipeline.R` (truncation parameters); full crosstabulation in Supplementary Table S14.

End of Supplementary Materials
